# Phenotypic and Functional Changes of Peripheral Ly6C^+^ T Regulatory Cells Driven by Conventional Effector T Cells

**DOI:** 10.3389/fimmu.2018.00437

**Published:** 2018-03-16

**Authors:** Jun Young Lee, Juhee Kim, Jaeu Yi, Daeun Kim, Hee-Ok Kim, Daehee Han, Jonathan Sprent, You Jeong Lee, Charles D. Surh, Jae-Ho Cho

**Affiliations:** ^1^Academy of Immunology and Microbiology, Institute for Basic Science, Pohang, South Korea; ^2^Department of Integrative Biosciences and Biotechnology, Pohang University of Science and Technology, Pohang, South Korea; ^3^Immunology Division, Garvan Institute of Medical Research, Darlinghurst, NSW, Australia; ^4^St Vincent’s Clinical School, University of New South Wales, Sydney, NSW, Australia; ^5^Division of Developmental Immunology, La Jolla Institute for Allergy and Immunology, La Jolla, CA, United States

**Keywords:** Ly6C, naive CD4 T regulatory cells, effector CD4 T regulatory, conventional effector T cells, T cell receptor, self-peptide/major histocompatibility complex ligands

## Abstract

A relatively high affinity/avidity of T cell receptor (TCR) recognition for self-peptide bound to major histocompatibility complex II (self-pMHC) ligands is a distinctive feature of CD4 T regulatory (Treg) cells, including their development in the thymus and maintenance of their suppressive functions in the periphery. Despite such high self-reactivity, however, all thymic-derived peripheral Treg populations are neither homogenous in their phenotype nor uniformly immune-suppressive in their function under steady state condition. We show here that based on the previously defined heterogeneity in the phenotype of peripheral Treg populations, Ly6C expression on Treg marks a lower degree of activation, proliferation, and differentiation status as well as functional incompetence. We also demonstrate that Ly6C expression on Treg in a steady state is either up- or downregulated depending on relative amounts of tonic TCR signals derived from its contacts with self-ligands. Interestingly, peripheral appearance and maintenance of these Ly6C-expressing Treg cells largely differed in an age-dependent manner, with their proportion being continuously increased from perinatal to young adult period but then being gradually declined with age. The reduction of Ly6C^+^ Treg in the aged mice was not due to their augmented cell death but rather resulted from downregulation of Ly6C expression. The Ly6C downregulation was accompanied by proliferation of Ly6C^+^ Treg cells and subsequent change into Ly6C^−^ effector Treg with concomitant restoration of immune-suppressive activity. Importantly, we found that this phenotypic and functional change of Ly6C^+^ Treg is largely driven by conventional effector T cell population. Collectively, these findings suggest a potential cross-talk between peripheral Treg subsets and effector T cells and provides better understanding for Treg homeostasis and function on maintaining self-tolerance.

## Introduction

CD4 T regulatory (Treg) cells are well defined for their potent immune-suppressive activity and establishment of self-tolerance, and are known to develop in the thymus as a result of positive selection from their thymic precursor cells (CD4^+^ CD8^+^ double-positive thymocytes) by relatively a high affinity/avidity of T cell receptor (TCR) interactions with self-peptide-major histocompatibility complex II (self-pMHC) ligands, which is referred to as thymic (or natural) Treg (tTreg) and constitutes a major population of peripheral Treg pools under steady state (1, 2). Despite such well-defined high self-reactivity, however, steady-state mature Treg populations in the peripheral lymphoid organs are not uniformly homogenous but rather heterogenous in their phenotype and function, which differ in the levels of activation-associated surface markers, such as CD44 and CD62L, and the degrees of immune-suppressive activity, with naive (CD44^lo^ CD62L^hi^) and effector phenotype (CD44^hi^ CD62L^lo^) exhibiting the least and strongest activity, respectively (3).

Physiological importance of the relatively high self-reactivity of tTreg has been demonstrated not only for their thymic development but also for their peripheral survival and function (3). It is, therefore, possible that the defined phenotypic and functional heterogeneity within peripheral Treg populations might relate to their relative difference in the extent of TCR signaling for which they receive from interactions with self-ligands under steady state. In this regard, a recent study demonstrated that there was indeed a difference in the dependency of TCR signaling from self-recognition on the survival between naive versus effector Treg populations and that the latter effector cells compared to the former readily disappeared in the absence of such self-driven tonic TCR signaling (3). Interestingly, this study also showed that despite the remaining naive Treg cells persisted in the absence of tonic TCR signaling, these cells were not functional and thus failed to control conventional T cell activation and proliferation, suggesting an impaired immune-suppressive capacity of naive Treg cells (3). Although these prior observations provide strong evidence for differential dependency of self-reactivity of Treg populations on their peripheral homeostasis, however, whether and how these features relate to contribute to peripheral Treg pool’s heterogeneity in their survival, maintenance, and immune-suppressive function has not been fully understood.

With respect to the surface markers allowing for identifying and characterizing Treg phenotypic heterogeneity in an unambiguous reliable manner, recent studies have shown that steady-state peripheral Treg pools can be subdivided based on their surface expression of Ly6C with far clearer distinction being made for their relative activation status and functional competence compared to the aforementioned combinations of CD44 and CD62L expression (4). In a steady-state condition, expression of Ly6C on CD4 T cell compartment has been shown to occur mainly in naive cells but under inflammatory conditions also found to be induced in effector cells (4–6). Moreover, the Ly6C expression on naive CD4 T cells was shown to be influenced by tonic TCR signals derived from self-recognition, with surface levels of Ly6C being inversely correlated with relative avidity of TCR interactions with self-ligands (4). Likewise, studies have also shown that Ly6C expression on peripheral Treg cell compartment was negatively regulated by such tonic TCR signals, low levels of which promote upregulation of Ly6C and generate Ly6C^+^ Treg population under steady state (4). Further characterizations with these Ly6C^+^ Treg cells have demonstrated that these cells, unlike their Ly6C^−^ counterparts, display a decreased level of Ki-67 expression, a poor immune-suppressive activity both *in vitro* and *in vivo*, and gradually disappeared with age, and thus made a conclusion that the steady-state peripheral Ly6C^+^ Treg cells are non-functional apoptosis-prone populations (4).

Given their known poor functionality of Ly6C^+^ Treg cells presumably with a minimal contribution to maintaining peripheral self-tolerance, it is questionable whether the existence of such pro-apoptotic and non-functional Treg subset may have any physiological significance. Furthermore, considering the nature of Treg of receiving relatively a strong TCR signal from self-interactions, it is still unclear how a certain fraction of Treg cells would reduce their high intrinsic self-reactivity just below a threshold level for responding to self-ligands and become quiescent naive Ly6C^+^ Treg subset. In this report, we show that steady-state Treg cells in the periphery compete for their niches where they sense and receive a different quantity and quality of self-ligands, and that the difference in the response strength for self-reactivity contributes to the generation of Ly6C^+^ Treg cells. We also show that the Ly6C^+^ Treg subset is a unique in their ability to downregulate Ly6C expression in a particular environment of aging or inflammation, and importantly, this change is accompanied by enhanced proliferation and transition into Ly6C^−^ effector Treg cells *via* a mechanism involving conventional effector T cells. These findings suggest that Ly6C^+^ Treg cells serve as a precursor that can switch into effector Treg pools and have a physiological role in balancing self-tolerance and immunity.

## Materials and Methods

### Mice

C57BL/6 (B6), Nur77-eGFP (7), Foxp3-eGFP (8), OT-II Rag1^−/−^ (9), H2M^−/−^ (10), Rag1^−/−^ (11), IL-2^−/−^ (12), Foxp3-DTR (13), and CD11c.DOG Rag1^−/−^ (14) mice were maintained at POSTECH Biotech Center (PBC, Korea) under specific pathogen-free (SPF) condition. Foxp3-eGFP knock-in mice were a gift from Dr. Talal Chatila (University of California at Los Angeles, Los Angeles, CA, USA) (8). Thymectomy was performed with Foxp3-eGFP mice as previously described (15). Germ-free (GF) and antigen-free (AF) mice are maintained at PBC as described (16). Unless it is specified, 6–12-week-old mice were used for the experiments according to the protocols approved by the Animal Experimental and Ethic Committee at the Institute for Basic Science (Korea).

### Flow Cytometry Analysis

Cell suspensions were prepared and stained for FACS analysis of cell-surface markers using PBS containing 2% FBS and 0.05% sodium azide with the following mAbs to (from BD Biosciences, Biolegend, and eBioscience): CD3 (145-2C11), CD4 (GK1.5 and RM4–5), CD5 (53-7.3), CD8α (53-6.7), CD11b (M1/70), CD11c (N418), CD19 (MB19-1), CD24 (M1/69), CD28 (37.51), CD43 (1B11), CD44 (IM7), CD45.1 (A20), CD45.2 (104), CD62L (MEL-14), CD69 (H1.2F3), CD90.1 (HIS51 or OX-7), CD25 (PC61), CD103 (2E7), bromodeoxyuridine (BrdU) (3D4), and Ly6C (HK1.4) in a conjugation with FITC, PE, PE-Cy5, PE-Cy7, APC, APC-Cy7, or PB. Propidium iodide (PI) was purchased from Sigma Aldrich. To detect dead cell in flow cytometry, PI was used at 500 ng/ml of final concentration for staining of 1–5 × 10^6^ of cells. Flow cytometry samples were run using a LSR II or FACSCanto II (BD Biosciences) and analyzed with FlowJo software (Tree Star).

### BrdU Uptake Analysis

Foxp3-eGFP mice were fed with 0.8 mg/ml of BrdU in drinking water for 10 days and the BrdU uptake level on Treg subsets were analyzed on day 11. BrdU staining was performed according to the manufacturer’s protocol (BD biosciences).

### Treg Subset Purification

Pooled lymph node (LN) cells from the Foxp3-eGFP mice were depleted for CD4^−^ cells by series of biotinylated antibody; CD8α, CD11b, CD11c, CD24, CD19, B220, and IMag according to the manufacturer’s protocol (BD biosciences). Enriched cells were stained with PI and fluorochrome-conjugated Abs to CD4, Ly6C, CD62L and CD44, and then sorted to obtain CD4^+^ eGFP^+^ and Ly6C^+^ CD62L^hi^, Ly6C^−^ CD62L^hi^, and Ly6C^−^ CD62L^lo^ populations. To harvest activated subset of Treg, sorted Treg subsets from Thy1.1^+^ Foxp3-eGFP mice were transferred into Foxp3-DTR mice with continuous host Treg depletion by diphtheria toxin (DT; Sigma Aldrich) treatments. After 2 weeks of Treg subset transfer, the transferred Treg cells were reenriched from the LN and spleen of the hosts by depleting CD45.2 (104) positive cells using magnetic bead. The enriched cells were sorted with using a Moflo-XDP (Beckman Coulter). Purity was routinely tested after cell sorting and was 95–99%. When Treg subsets were transferred into lympho-replete host, the donor Treg cells were enriched by biotinylated anti-Thy1.1 (HIS51) and magnetic beads. After enrichment of donor cells, Thy1.1 was stained with fluorochrome-conjugated anti-Thy1.1 (OX7) before FACS analysis.

### Mixed Bone Marrow (BM) Chimera Generation

Bone marrow cells from CD11c.DOG Rag1^−/−^ were mixed with BM cells from wild-type (WT) Rag1^−/−^ mice at the indicated ratio and then this mixture of BM cells (50%) were mixed again with BM cells from OT-II Thy1.1^+^ Foxp3-eGFP Rag1^−/−^ mice (50%). These BM mixtures (5 × 10^6^) were injected into irradiated Rag1^−/−^ hosts (300 cGy). At 8 weeks after BM cell transfer, cells from LN and spleen were analyzed by flow cytometry for Ly6C expression and CD5.

### Cell Tracker Violet (CTV) Labeling and *In Vitro* Proliferation

FACS-purified subsets of Treg from Foxp3-eGFP mice were labeled with CTV (Thermo Fisher Scientific) according to the protocol from manufacturer. CTV-labeled Treg cells were cultured (5 × 10^4^/well) for 3 days with plate-bound anti-CD3 (5 µg/ml) plus indicated cytokines including rmIL-2, rmIL-4, rmIL-9, rmIL-21, and TNF-α (all 10 ng/ml). All cytokines were purchased from Peprotech.

### Ly6C Upregulation on Recently Developed Thymic Treg

To assess Ly6C upregulation by recently developed Treg in response to self-antigen competition, thymocytes from 3- to 4-week-old Foxp3-eGFP mice were enriched for CD8^−^ cells by depleting CD8^+^ cells using magnetic beads. The enriched CD8^−^ thymocytes were labeled with CTV and then indicated numbers of cells were injected into irradiated B6 hosts (600 cGy).

### Tconv Cell-Dependent Proliferation of Treg^N^Ly6C^+^ Treg

T regulatory subsets and naive CD4 T cells were prepared as described above. Treg subsets (5 × 10^5^) were injected into Rag1^−/−^ hosts which rose under GF condition. To assess the role of conventional T cells (Tconv) on Treg proliferation, 1 × 10^6^ of naive CD4 T cells were coinjected where indicated.

### Adoptive Transfer into Foxp3-DTR Host with DT Treatment

Wild-type or Foxp3-DTR mice were treated with 1 µg of DT intraperitoneally in every other day or every third day as indicated. To measure proliferation of donor Treg subsets in Treg-depleted host, ~3–5 × 10^5^ of CTV-labeled Treg subsets were injected into WT or Foxp3-DTR hosts.

### IL-2 Complex Treatment

1:5 molecular ratios of rmIL-2 and anti-mouse IL-2 (JES6-1) (Biolegend) were preincubated (30 min at room temperature). The rmIL-2/JES6-1 complex (1/5 μg) was daily injected intraperitoneally for on days 0–2. To measure proliferation of donor Treg subsets in response to high concentration of IL-2, 5 × 10^5^ of CTV-labeled Treg subsets were injected into WT hosts.

### Statistical Analysis

Results represent the mean ± SEM unless indicated otherwise. Statistical significance was determined by the unpaired Student’s *t*-test. Statistical analyses were performed using Prism GraphPad software v5.0 (*p < 0.05; **p < 0.01; ***p < 0.001).

## Results

### Phenotypic Heterogeneity in Peripheral Treg Population

Given the recently defined phenotypic and functional heterogeneity of peripheral CD4 Treg populations (4, 17), we sought to address whether and how these features relate to their TCR reactivity to self-pMHC ligands. We thus first characterized phenotypes of peripheral CD4 Treg using Foxp3-GFP reporter mice based on the expression of various cell surface markers characteristics of naive or activated effector/memory cells.

**Figure 1:**
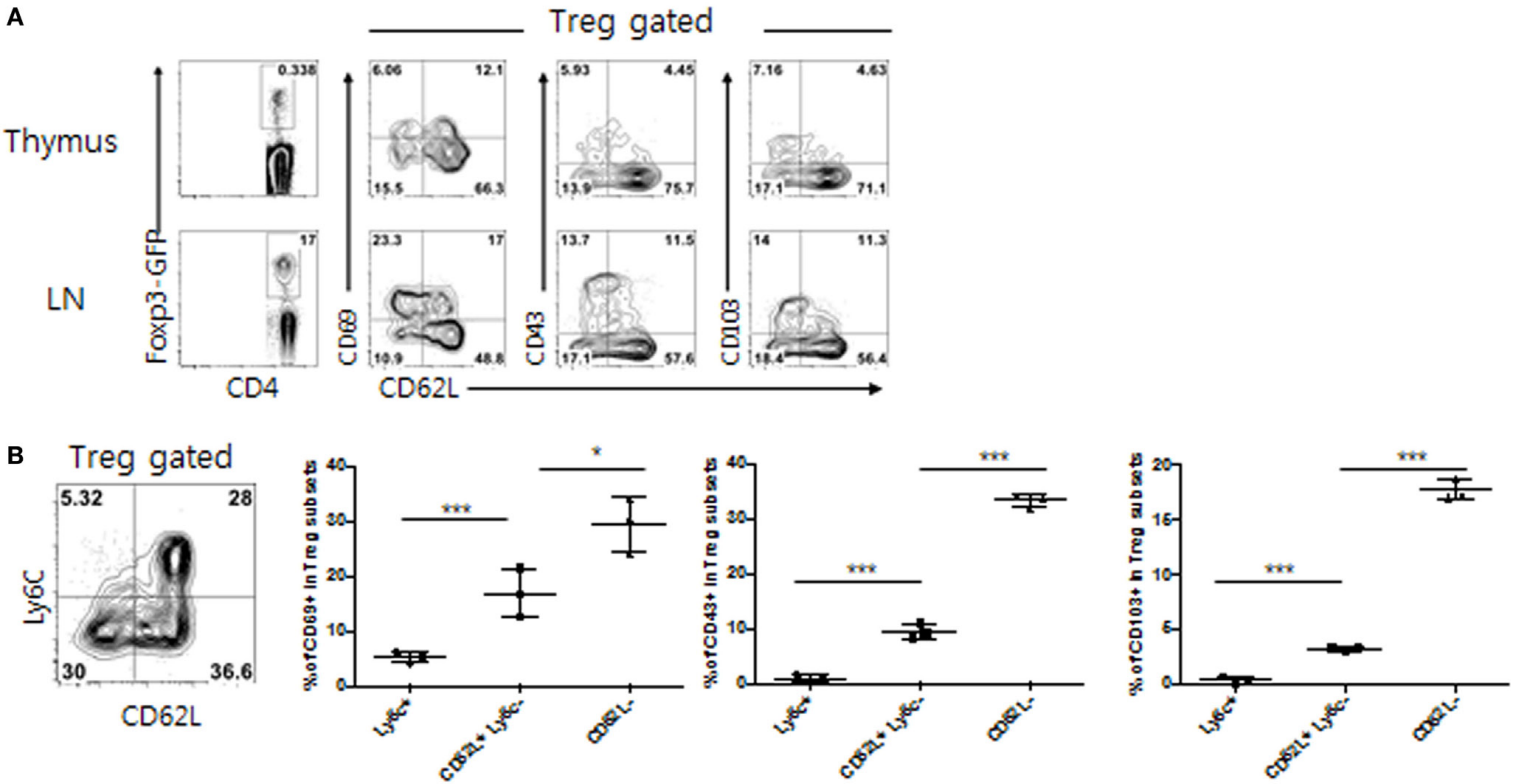
Phenotypic heterogeneity of peripheral T regulatory (Treg) populations. **(A)** Lymphocytes from thymus or peripheral lymph nodes (PLN) from Foxp3-eGFP mice were analyzed for indicated marker expression. **(B)** Treg subsets from PLN of Foxp3-eGFP mice were analyzed for CD69, CD103, and CD43 expression. The data are representative of three experiments comprising three mice per group. *p < 0.05, **p < 0.01, and ***p < 0.001.

As shown in Figure 1A, within the peripheral LNs, but not thymic, CD4^+^ Foxp3^+^ Treg populations, several distinct subsets were identifiable mostly based on the expression of CD62L in combination with effector-associated markers CD69, CD43, and CD103. While subsets expressing low levels of CD62L (CD62L^lo^) exhibited more activated phenotypes (higher levels of CD69, CD43, and CD103), CD62L^hi^ subsets appeared to constitute a naive pool, although ~15–25% of these CD62L^hi^ subsets were still, albeit lower than those of CD62L^lo^ subsets, able to express those activation markers. The clearest phenotypic distinction, however, was obtained with the expression of Ly6C, resulting in the separation of peripheral Treg cells into largely three distinct subpopulations: CD62L^lo^ Ly6C^–^, CD62L^hi^ Ly6C^–^, and CD62L^hi^ Ly6C^+^ (Figure 1B), subsets of which have also been well characterized by previous studies (18) Consistent with those of prior observations, the former subset (CD62L^lo^ Ly6C^–^) showed an activated/effector phenotype with highest levels of CD69, CD43 and CD103 expression (denoted as Treg^Eff^), whereas the latter two subsets (CD62L^hi^ Ly6C^−^ and CD62L^hi^ Ly6C^+^) exhibited a naive phenotype with either intermediate or lowest amounts of these markers (denoted as Treg^N^ Ly6C^−^ and Treg^N^ Ly6C^+^, respectively) (Figure 1B). These data therefore confirmed the previous notion that peripheral Treg cells, large majority of which is thymically derived, neuropilin-1-expressing natural Treg (data not shown), are heterogenous and largely differ in their cellular activation states from one subset to another.

### Influence of Variable Quantities of TCR Self-Recognition on Ly6C^+^ Naive Treg Generation

The difference in the extent of activation state of peripheral Treg pools is intriguing as these cells are known to have a relatively strong intrinsic TCR affinity for self-pMHC ligands (2, 17), the affinity levels of which is perhaps sufficient to cause a uniform, homogenous pattern of effector Treg populations. However, the above results showing peripheral existence of a naive state of Treg cells raise a possibility that each subset of Treg may differ in their relative TCR strength of self-recognition and that the degree of such TCR signaling may be quantitatively or qualitatively lower for naive subsets, particularly Treg^N^ Ly6C^+^ cells, than those of effector (Treg^Eff^) subset, perhaps limiting the peripheral abundancy of the former subsets. The possibility of differential degree of TCR self-recognition was indeed supported by moderately lower expression of CD5 and Nur77, levels of which are known to correlate with the strength of TCR affinity for self-pMHC ligands (7), in Treg^N^ Ly6C^+^ subset than those of both Treg^N^ Ly6C^−^ and Treg^Eff^ subsets (Figure S1A in Supplementary Material). This was also in line with a modest reduction in the basal phosphorylation of CD3ζ chain, indicative of the extent of TCR self-reactivity, in Treg^N^ Ly6C^+^ subset compared to Ly6C^−^ subsets as reported previously (4).

**Figure 2:**
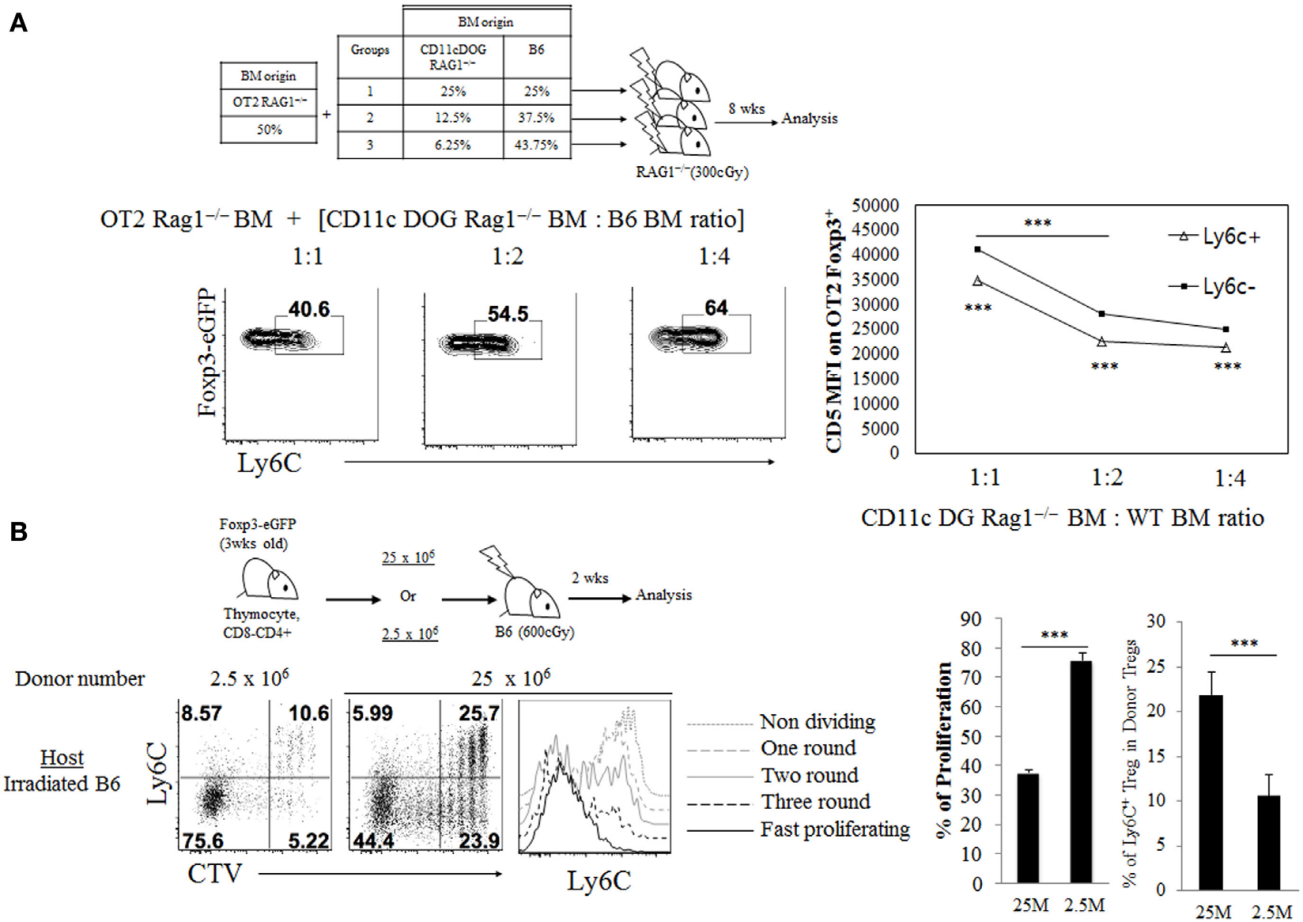
Impact of variable quantities of T cell receptor self-recognition on Treg^N^ Ly6c^+^ subset generation. **(A)** Bone marrow (BM) cells from CD11c.DOG Rag1^−/−^ were mixed with BM cells from wild-type WT Rag1^−/−^ mice at the indicated ratio and then this mixture of BM cells (50%) were mixed again with BM cells from OT-II Thy1.1^+^ Foxp3-eGFP Rag1^−/−^ mice (50%). These BM cell mixtures were injected into irradiated (300 cGy) Rag1^−/−^ hosts. At 8 weeks after BM cell transfer, cells from peripheral lymph node (PLN) were analyzed by flow cytometry for Ly6C expression and CD5. Shown are gated on Thy1.1^+^ CD4^+^ Foxp3^+^ cells. **(B)** Cell tracker violet (CTV) labeled, indicated number of CD8-depleted thymocytes from Thy1.1^+^ Foxp3-eGFP mice were injected into irradiated B6 hosts and then proliferation and Ly6C expression were analyzed on day 14 after transfer. Shown are proliferation and the Ly6C expression on donor Thy1.1^+^ Foxp3^+^ CD4^+^ cells in PLN. The data are representative of three experiments comprising four to five mice per group. *p < 0.05, **p < 0.01, and ***p < 0.001.

We next examined whether such subtle difference in TCR signaling derived from self-recognition contributes to generating naive Treg, especially Treg^N^ Ly6C^+^ cells, and determining their overall size in the periphery under steady state. To address this in an unbiased system, we first used monoclonal OT-II TCR transgenic system specific for MHC-II-restricted ovalbumin (Ova) antigen and generated mixed BM chimera. For BM donors, OT-II.Rag1^−/−^ BM cells were combined with a titrated number of mixed BM cells from CD11c DOG.Rag1^−/−^ mice—which encode *Ova* transgene under control of CD11c promoter (14) and express Ova in dendritic cells (DCs) as a cognate self-antigen—and from WT B6 mice, and then intravenously (i.v.) injected into irradiated Rag1^−/−^ hosts (Figure 2A, top). In these BM chimeras, thymic development of OT-II Treg would require high affinity interaction of OT-II TCR with Ova peptide bound to MHC-II in the thymus, presumably with variable efficacy depending on the relative amounts of Ova self-antigen presented by DC derived from CD11c DOG BM. At 8 weeks after BM transfer, we indeed found the variable frequency of peripheral OT-II Treg generation, with the frequency being higher at 1:2 ratio than at 1:4 (and also 1:8) ratio of CD11c DOG BM to WT BM (Figure S2A in Supplementary Material). In contrast to the frequency of total peripheral OT-II Treg, however, the reverse applied to the frequency of Ly6C-expressing naive OT-II Treg generation (Figure 2A, bottom left); thus, the frequency of Treg^N^ Ly6C^+^ OT-II cells was far greater at 1:4 ratio than at 1:1 and 1:2 ratio of CD11c DOG BM to WT BM. Notably, the higher frequency of Ly6C^+^ Treg generation was associated with their low expression of CD5, whereas the lower generation was associated with the high expression of CD5 (Figure 2A, bottom right). These results suggest strong inverse correlation between the efficacy of Ly6C expression on Treg and the amounts of self-pMHC ligands present *in vivo*.

To further confirm such inverse relationship between the amounts of TCR self-recognition and generation of Treg^N^ Ly6C^+^ subset, we next used an adoptive transfer of polyclonal repertoire of thymic Treg precursor cells into lymphopenic hosts. For this, either a small or large number of CD4^+^ CD8^−^ thymocytes (that are Ly6C^–^; 25 × 10^6^ or 2.5 × 10^6^, respectively) were injected i.v. into irradiated B6 hosts (Figure 2B, top), in which the donor cells undergo lymphopenia-induced homeostatic proliferation (LIP) in a manner dependent on TCR self-interaction. At 14 days after transfer, several rounds of Treg cell proliferation were observed in both groups; however, the extent of proliferation was much greater with transfer of 2.5 × 10^6^ donor cells than transfer of 25 × 10^6^ donor cells, as assessed by CTV dye dilution (Figure 2B, bottom left) and expression of activation markers CD43 and CD62L (Figure S2B in Supplementary Material). In sharp contrast to the proliferative responses, the extent of Treg^N^ Ly6C^+^ cell generation was far less with transfer of 2.5 × 10^6^ donor cells than transfer of 25 × 10^6^ donor cells (Figure 2B, bottom right). These data suggest that adoptive transfer of smaller numbers of thymocytes results in less TCR competition for self-ligands, thereby inducing more intense TCR self-recognition and much lower efficacy of Ly6C^+^ Treg generation. Together, these results strongly support the idea that the generation of peripheral naive Treg^N^ Ly6C^+^ subset actively involves the relatively lower quantity of TCR signaling derived from contacts with self-pMHC ligands.

### Poor Responsiveness of Ly6C^+^ Naive Treg Subset to Tonic TCR Stimuli

**Figure 3:**
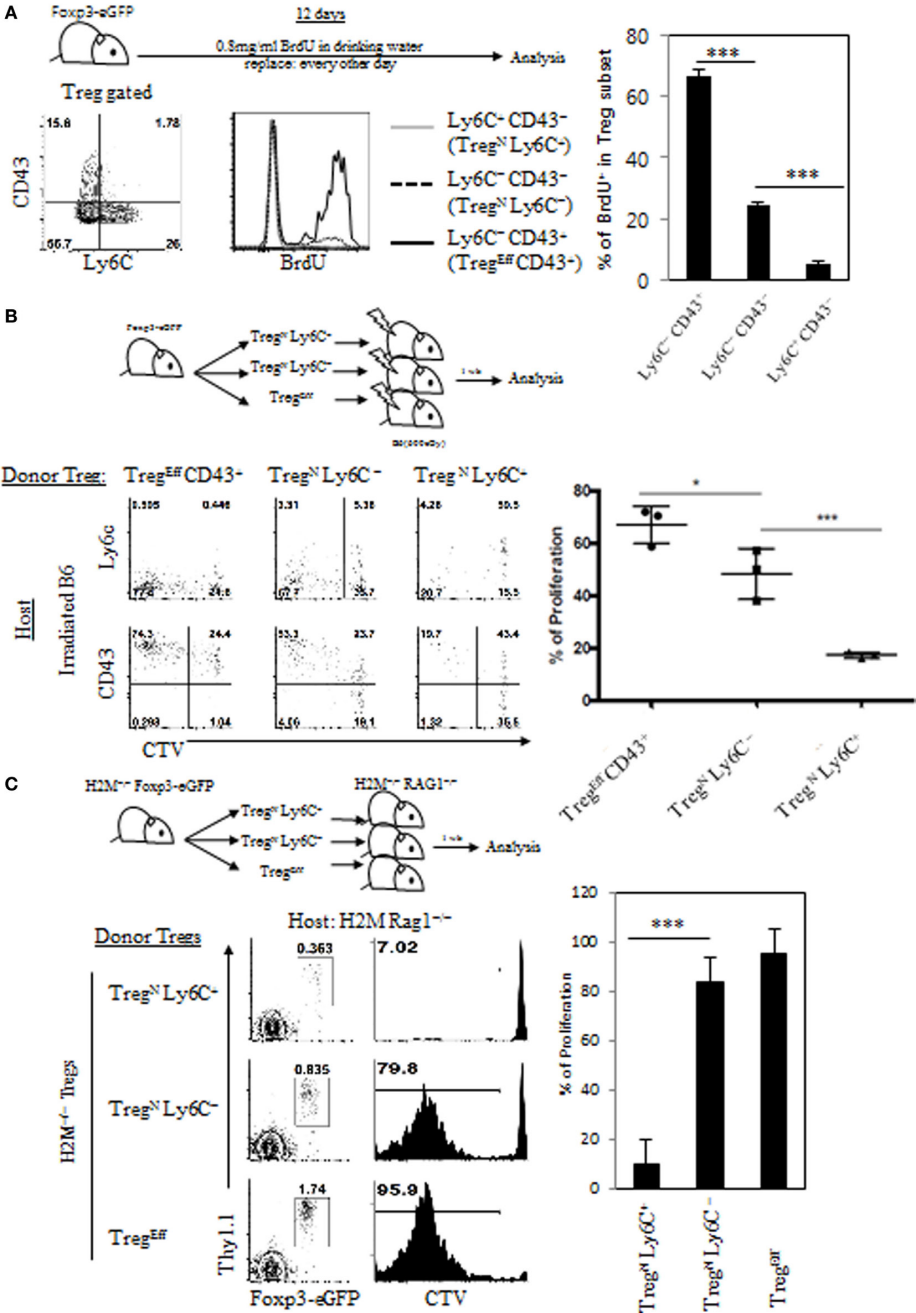
Poor responsiveness of Treg^N^ Ly6C^+^ subset to tonic T cell receptor stimuli. **(A)** Foxp3-eGFP mice were fed with 0.8 mg/ml of bromodeoxyuridine (BrdU) in drinking water for 10 days and then BrdU uptake levels on different subsets of T regulatory (Treg) were analyzed on day 10. Cell tracker violet-labeled Treg subsets from Thy1.1^+^ Foxp3-eGFP mice **(B)** or Thy1.1^+^ Foxp3-eGFP H2M^−/−^ mice **(C)** were injected into irradiated B6 **(B)** or H2M^−/−^ Rag1^−/−^ **(C)** hosts and then proliferations of donor Treg cells were analyzed on day 7 after transfer. The data are representative of three to four experiments comprising four to five mice per group. *p < 0.05, **p < 0.01, and ***p < 0.001.

Given the requirement of relatively lower intensity of TCR self-recognition for inducing Treg^N^ Ly6C^+^ cells, we next examined whether and how this feature relates to their functional responsiveness. In this regard, previous studies have shown that the Ly6C^+^ Treg cells exhibit lower levels of intracellular Ki-67 expression, a marker indicative of cell proliferation, compared to those of Ly6C^−^ Treg cells (4). We therefore sought to address whether such poor proliferative capacity of Ly6C^+^ Treg is a simple reflection of their activation status as a resting naive pool or a direct consequence of their lower degree of TCR signaling. To address this, we first analyzed *in vivo* proliferation of Treg subsets by feeding Foxp3-GFP reporter mice with BrdU for 10 days in drinking water (Figure 3A, top). In agreement with the previous report (4), effector Treg subset (Ly6C^−^ CD43^+^) exhibited a substantial degree of proliferation, whereas naive Treg subsets, especially Ly6C^+^ CD43^−^ cells, almost failed to do so, although Ly6C^−^ CD43^−^ cells showed only a little proliferation (Figure 3A, bottom). The data therefore support again the prior notion of the close relationship between Treg activation status and their functional responsiveness.

Based on the abovementioned result, we next examined whether the lack of *in vivo* proliferation of Ly6C^+^ Treg subset results from their lower quantity of TCR self-recognition under steady state condition. To this end, we adoptively transferred FACS-purified peripheral Treg donor subsets (i.e., Treg^Eff^ CD43^+^, Treg^N^ Ly6C^–^, and Treg^N^ Ly6C^+^; Figure S3A in Supplementary Material) into lymphodeplete irradiated B6 hosts (Figure 3B, top), in which the relative intensity of TCR signaling driven by self-recognition rises substantially to drive LIP of Treg cells (Figure 2B). At day 7 after adoptive transfer, as expected, Ly6C^−^ subsets of both effector and to a lesser extent naive cells exhibited significant levels of proliferative responses (Figure 3B, bottom); interestingly, however, this response was largely abrogated in Treg^N^ Ly6C^+^, even though a significant proportion of this Treg^N^ Ly6C^+^ subset exhibited signs of cellular activation such as upregulation of CD43 and downregulation of Ly6C. We also confirmed these findings by adoptive transfer of the above FACS-purified Treg subsets into unmanipulated lymphoreplete B6 hosts (Figures S3B,C in Supplementary Material) These data suggest that the Treg^N^ Ly6C^+^ subset, unlike its Ly6C^−^ naive counterparts, is somewhat refractory to tonic TCR stimuli both in a normal lymphoreplete and even in a lymphodeplete condition, with poor *in vivo* proliferative responses.

We then examined whether the refractory nature of tonic TCR signaling of Treg^N^ Ly6C^+^ subset is due to the ignorance of their TCR to an array of peripheral cognate self-pMHC ligands. This issue is of particular interest as this Treg^N^ Ly6C^+^ subset has been shown to display a skewed TCR repertoire compared to both Ly6C^−^ Treg^N^ and Treg^Eff^ subsets (18). To address this, we used Treg subsets purified from H2M^−/−^ mice that present only a single CLIP peptide bound to MHC-II molecules (CLIP-pMHC) in both thymus and periphery, generating limited TCR repertoire diversity (10). FACS-purified H2M^−/−^ Treg subsets (Thy1.1^+^) were adoptively transferred into H2M^−/−^ recipient hosts on a Rag1-deficient background (H2M^−/−^ Rag1^−/−^) (Figure 3C, top), in which donor Treg cells would undergo LIP dependent on tonic TCR signaling *via* its uniform recognition of a single self-antigen, CLIP-pMHC ligands. At day 7 after adoptive transfer, Ly6C^−^ subsets of both naive and effector H2M^−/−^ Treg cells exhibited a substantial degree of LIP, whereas Treg^N^ Ly6C^+^ cells failed to undergo LIP (Figure 3C, bottom), which was consistent with the results obtained from irradiated B6 hosts (Figure 3B). Together, all these results strongly suggest that Treg^N^ Ly6C^+^ cells are indeed a poor responder to tonic TCR stimuli, regardless of quantitative and even qualitative difference in any given self-pMHC ligands, and perhaps have a unique TCR tuning mechanism to maintain their functional quiescence under steady state condition.

### Age-Dependent Conversion from Treg^N^ Ly6C^+^ to Treg^Eff^ Subset

**Figure 4:**
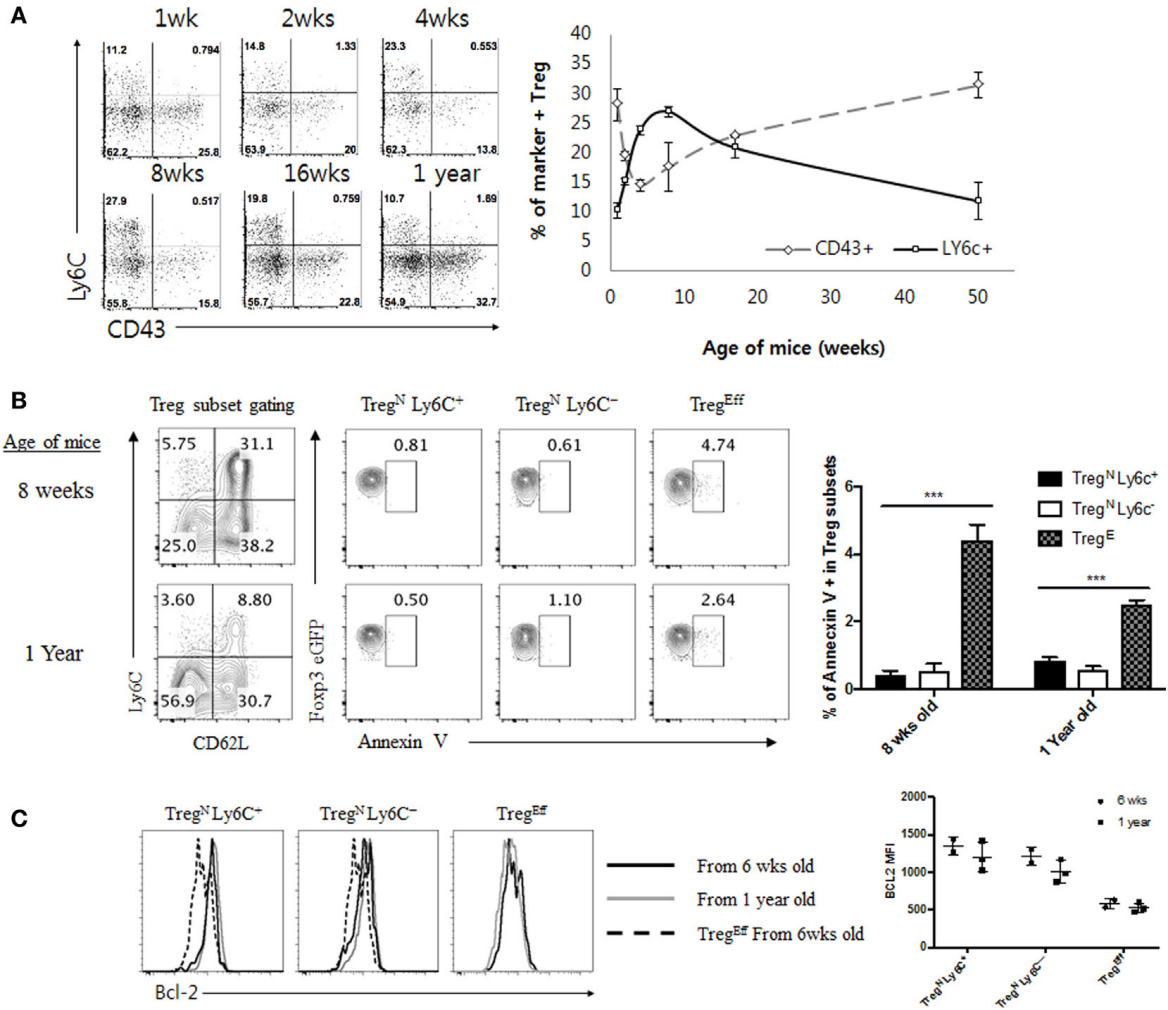
Age-dependent disappearance of Treg^N^ Ly6C^+^ is not correlated with increased apoptosis in aged mice. **(A)** Peripheral lymph node cells from Foxp3-eGFP mice of various ages indicated were stained with CD43, CD4, Ly6C, and PI. Shown are CD43 and Ly6C expression on CD4^+^ eGFP^+^ cells (left) and statistics (right). **(B)** T regulatory (Treg) subsets from young (8 weeks) or aged (1 year) mice Foxp3-eGFP mice were stained with Annexin V, CD4, PI, Ly6C, and CD62L. Shown are gated on PI^−^ CD4^+^ eGFP^+^ Treg (top left) and then gated on indicated markers. **(C)** Treg subsets from young (8 weeks) or aged (1 year) mice Foxp3-eGFP mice were analyzed for Bcl-2 expression. The data are representative of three experiments comprising two to three mice per group. *p < 0.05, **p < 0.01, and ***p < 0.001.

Given the poor responsiveness of Treg^N^ Ly6C^+^ subset to tonic TCR stimuli, this subset may not be able to persist in the periphery for a longer period of time under steady state. In order to overcome this, it is possible that Treg^N^ Ly6C^+^ cells may depend on additional cues other than self-ligands for their survival and maintenance. In fact, relatively a shorter life-span of the Treg^N^ Ly6C^+^ subset was reported previously, showing a uniform reduction of this subset in aged mice, with enhanced levels of their apoptosis compared to those of Ly6C^−^ Treg subset (4). Likewise, in a time course analysis for naive (Ly6C^+^) versus effector (CD43^+^) Treg subset, we found the increase in the frequency of the Treg^N^ Ly6C^+^ subset during perinatal to a young adult period, with reaching a peak at ~8–10 weeks of age, but then uniform gradual decrease afterward over 50 weeks of age (Figure 4A). Similar data were also obtained in the absolute number of these Treg subsets with age (data not shown). Survival differences between Ly6C^+^ and Ly6C^−^ Treg subsets may account for this phenomenon. The age-dependent decline of Ly6C^+^ subset, however, was not a result of their increased apoptotic death, as assessed by the reduced amounts of Annexin V staining for naive Treg^N^ Ly6C^+^ (and also Treg^N^ Ly6C^−^) subset compared to those of Treg^Eff^ subset (Figure 4B); here, the lower levels of apoptosis of Treg^N^ subsets appeared to be similar for both young (8-week-old) and old (1-year-old) mice. Moreover, consistent with the diminished apoptosis, Treg^N^ subsets—regardless of age—exhibited higher expression of anti-apoptotic protein Bcl-2 than Treg^Eff^ cells (Figure 4C), which was also in close agreement with a recent report showing a higher level of Bcl-2 for Ly6C^+^ naive than for Ly6C^−^ effector Treg (18). Based on these data showing lower *ex vivo* apoptosis and higher Bcl-2 expression, we further confirmed the survival difference of Treg subsets *in vitro* by measuring spontaneous cell death in culture (Figure S4A in Supplementary Material); again, the results clearly showed much less apoptotic death in Treg^N^ subsets than in Treg^Eff^.

The gradual reduction of Treg^N^ Ly6C^+^ subset in the aged mice despite their enhanced ability for survival suggests an existence of additional mechanism by which this subset is actively removed in an age-dependent manner. Phenotypic conversion from Ly6C^+^ into Ly6C^−^ Treg subset *via* downregulation of Ly6C expression on the former was the most plausible explanation with the following two observations. First, levels of Ly6C were decreased substantially when Treg^N^ Ly6C^+^ cells were stimulated by TCR ligation with anti-CD3 and anti-CD28 antibodies *in vitro* (Figure S4B in Supplementary Material), which was consistent with previously reported findings *in vitro* (4) and our prior adoptive transfer data with this subset in lymphopenic hosts *in vivo* (Figure 3B). Second, the age-dependent change of the peripheral frequency of Treg^Eff^ CD43^+^ subset was in sharp contrast to those of Treg^N^ Ly6C^+^ subset, with the proportion of the former being decreased during perinatal to the young adult period but then being gradually increased thereafter with age (Figure 4A). Besides the age-dependent frequency change, it should be noted that Treg^Eff^ cells from young vs. aged mice showed similar levels of expression of various Treg surface markers (i.e., CD43, CD62L, CD103, GITR, and ICOS), except for CD69, CTLA4, and PD-1 (Figure S4C in Supplementary Material and data not shown). These data together support the idea of an age-dependent transition from Treg^N^ Ly6C^+^ to Treg^Eff^ subset.

**Figure 5:**
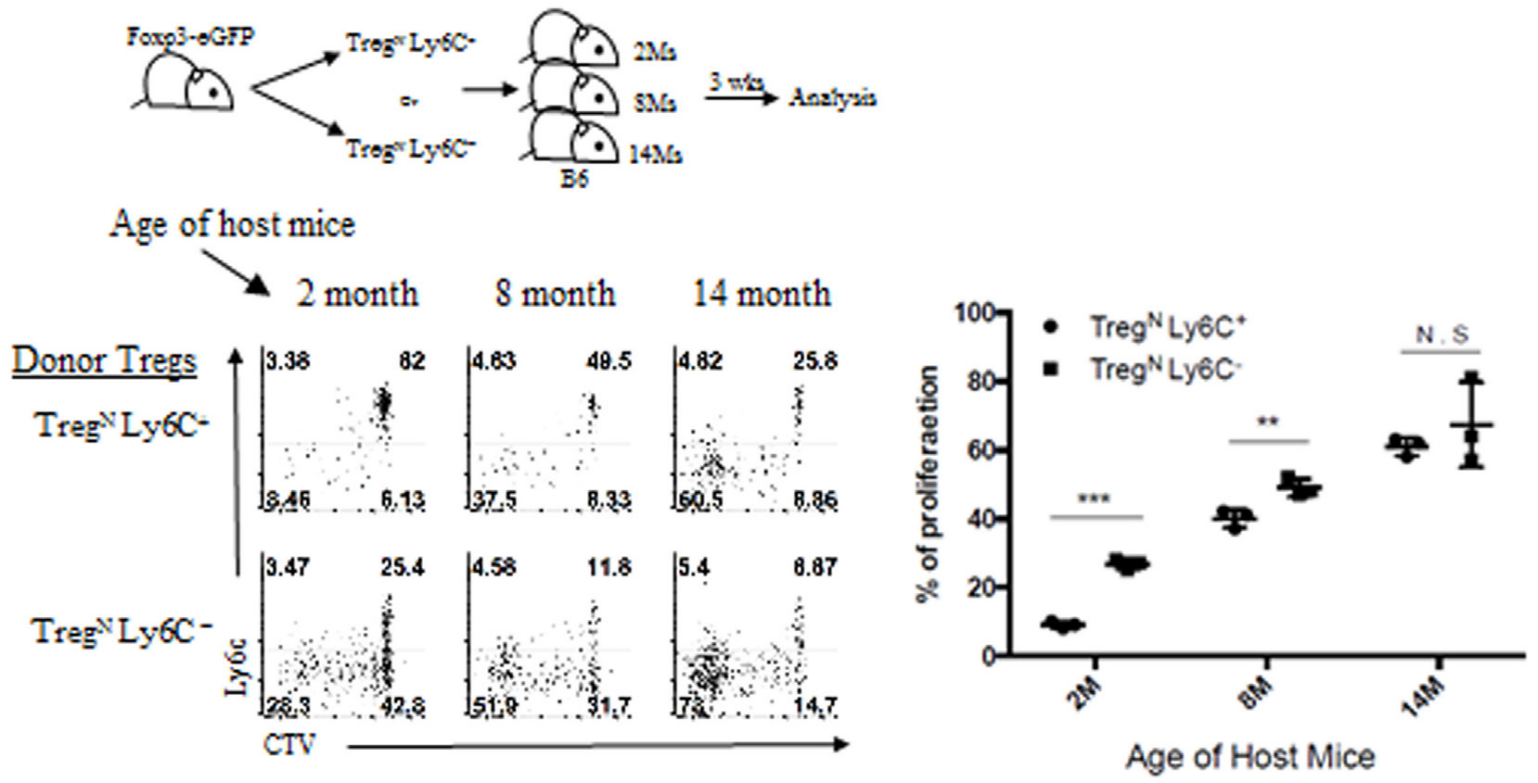
Age-dependent conversion of Treg^N^ Ly6C^+^ subset into Treg^Eff^. T regulatory (Treg) subsets from Thy1.1 + Foxp3-eGFP were labeled with cell tracker violet and then injected into differentially aged hosts. Donor cell proliferation was analyzed 3 weeks after transfer. The data are representative of three experiments comprising three to five mice per group. *p < 0.05, **p < 0.01, and ***p < 0.001.

We then examined the underlying mechanisms of Treg phenotypic conversion in aged mice. To this end, we first sought to define essential factor(s) that promotes such age-dependent conversion and tested a possibility of whether this phenomenon is attributed to enhanced activation of Treg^N^ Ly6C^+^ subset presumably *via* occasional increase of foreign environmental antigens in aged mice housed under SPF condition. This possibility, however, was not the case, as the enhanced frequency of Treg^Eff^ subset (along with reduced frequency of Ly6C^+^ Treg subset) was similarly observed both in aged SPF mice and in aged GF and even dietary AF mice (data not shown). This finding seems to rule out a role of innocuous foreign antigens, such as commensal microbiota and foods, yet highlights a possible involvement of self-antigens perhaps in conjunction with other age-dependent homeostatic cues. We thus further assessed whether there are any age-dependent cues that can facilitate the phenotypic conversion of Treg^N^ Ly6C^+^ subset. To address this, we adoptively transferred either FACS-purified Ly6C^+^ or Ly6C^−^ naive (CD62L^hi^ CD43^−^) Treg subset into recipient mice of different ages (ranging from 2 to 14 months of age; Figure 5, top). At 3 weeks after transfer into 2-month-old young recipient mice, as expected, a large majority of Treg^N^ Ly6C^+^ cells compared to Ly6C^−^ Treg cells were barely dividing with only about ~8% of cells proliferating and downregulating Ly6C (Figure 5, bottom). Notably, however, the proportion of these responses significantly increased up to ~40% with adoptive transfer into 8-month-old recipient mice, and even further increased up to ~65% after transfer into 14-month-old recipient mice, a level of which was near identical to those seen after transfer with Ly6C^−^ Treg subset into these aged hosts (Figure 5, bottom right).

Together, these data strongly suggest that while the responsiveness of Treg^N^ Ly6C^+^ subset to tonic TCR stimuli is actively suppressed under steady-state environment of young adult mice, this subset in older mice may have a unique ability to sense a certain age-dependent cue and switch from naive to effector phenotype.

### Role of Conventional Effector T Cells in the Age-Dependent Treg Conversion

In an attempt to define the nature of an age-dependent homeostatic cue that promotes most likely Treg^N^ Ly6C^+^ conversion into Treg^Eff^, we determined if such conversion is correlated with the large increase in conventional effector/memory CD4 T cell populations (Tconv^Eff^) which are known to be generated spontaneously under steady state and gradually increased in number with age (19). In comparison of these cells from mice at different ages, their frequencies in the spleen were indeed largely increased in older mice (12-month-old and to a lesser extent 4 months old; ~42 and 30%, respectively) compared to those of young mice (2-month-old; ~18%), with a conventional CD44^hi^ CD62L^lo^ profile characteristic of effector/memory phenotype (Figure S5A in Supplementary Material), perhaps correlating to the enhanced conversion of Treg^N^ Ly6C^+^ to Treg^Eff^ in the older mice (Figure 5). Likewise, the similar relationship between the increase of Tconv^Eff^ cells and augmented Treg conversion from naive to Treg^Eff^ was also observed in aged thymectomized mice, in which CD4 Tconv^Eff^ pools (CD43^+^ Ly6C^−^) are known to increase (Figure S5B in Supplementary Material). Thus, peripheral Tconv^Eff^ cells in 9-month-old thymectomized mice were observed with ~4-fold increase in their frequency relative to those seen in sham-thymectomized mice, correlating with enhanced Treg conversion from Treg^N^ Ly6C^+^ to Treg^Eff^ CD43^+^ subset in the former (Figure S5B in Supplementary Material).

**Figure 6:**
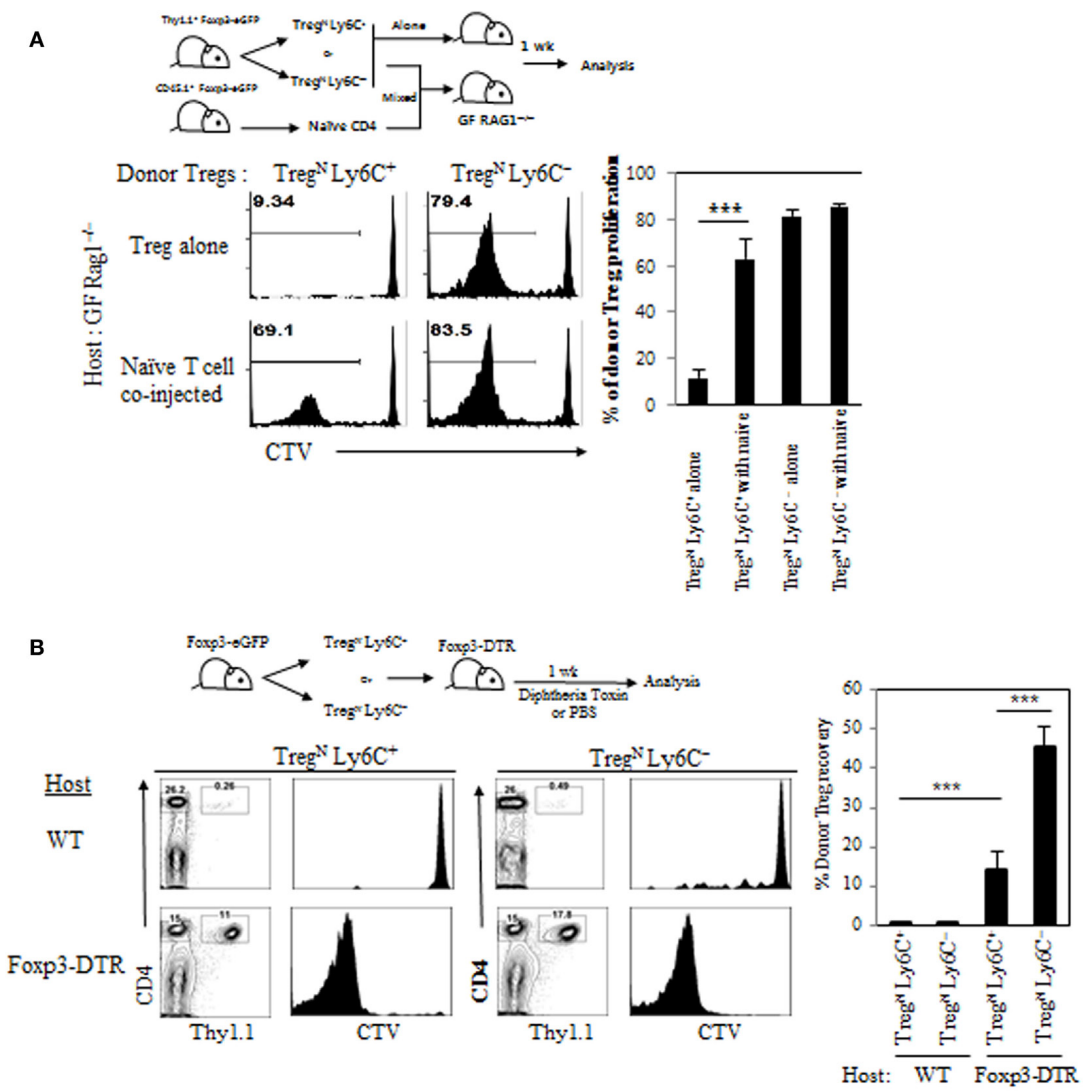
Role of Tconv^Eff^ cells in functional restoration of Treg^N^ Ly6C^+^ cells. **(A)** T regulatory (Treg) subsets from Thy1.1^+^ Foxp3-eGFP mice were labeled with cell tracker violet (CTV) and then injected into germ-free Rag1^−/−^ hosts in the presence or absence of naive CD4 T cells. Shown are proliferations of donor Treg subsets on day 7. **(B)** Treg subsets from Thy1.1^+^ Foxp3-eGFP mice were labeled with CTV and then injected into wild type or Foxp3-DTR hosts. The hosts were treated with 1 µg of diphtheria toxin in every other day. Donor cell proliferation was analyzed on day 7 after transfer. The data are representative of three to four experiments comprising three to four mice per group. *p < 0.05, **p < 0.01, and ***p < 0.001.

To gain more direct evidence supporting the roles of Tconv^Eff^ populations in contributing to the Treg^N^ Ly6C^+^ conversion, we used two different approaches that can generate increased amounts of Tconv^Eff^ cells at variable degrees depending on different immune environment of experimental mice being engaged. First, we adoptively transferred either Treg^N^ subset (either Ly6C^+^ or Ly6C^−^) alone or a mixture of Treg and naive CD4 T cells into GF Rag1^−/−^ hosts (Figure 6A, top); here, the latter cotransferred naive CD4 T cells undergo moderate but significant activation and proliferation to differentiate into functional Tconv^Eff^ cells (data not shown) (20). At day 7 after adoptive transfer, Treg^N^ Ly6C^−^ subset underwent similar degree of robust proliferation, regardless of whether recipient mice were coinjected with naive CD4 T cells (Figure 6A, bottom). Importantly, however, Treg^N^ Ly6C^+^ subset, although failed to proliferate alone, showed significantly increased levels of proliferation only with the aid of cotransferred naive CD4 T cells (Figure 6A, bottom). Second, we adoptively transferred Treg^N^ Ly6C^+^ or Treg^N^ Ly6C^−^ subsets into either WT or Foxp3-DTR (DT receptor) mice (Figure 6B, top), in which depletion of the host Foxp3^+^ Treg by DT injection results in robust activation/expansion of host naive CD4 T cells and generate Tconv^Eff^ populations (13). At day 7 after adoptive transfer and DT treatment, donor Treg^N^ Ly6C^+^ subset showed no proliferation, although Treg^N^ Ly6C^−^ subset proliferated moderately in WT hosts (Figure 6B, bottom). In marked contrast, however, Treg^N^ Ly6C^+^ subset showed vigorous proliferation comparable to those of Treg^N^ Ly6C^−^ subset in Foxp3-DTR hosts (Figure 6B, bottom). Together, these findings strongly suggest that Tconv^Eff^ cells play a critical role in converting Treg^N^ Ly6C^+^ subset to become Treg^Eff^ subset.

### Role of IL-2 in the Effector Conversion of Ly6C^+^ Treg Subset and their Proliferation

**Figure 7:**
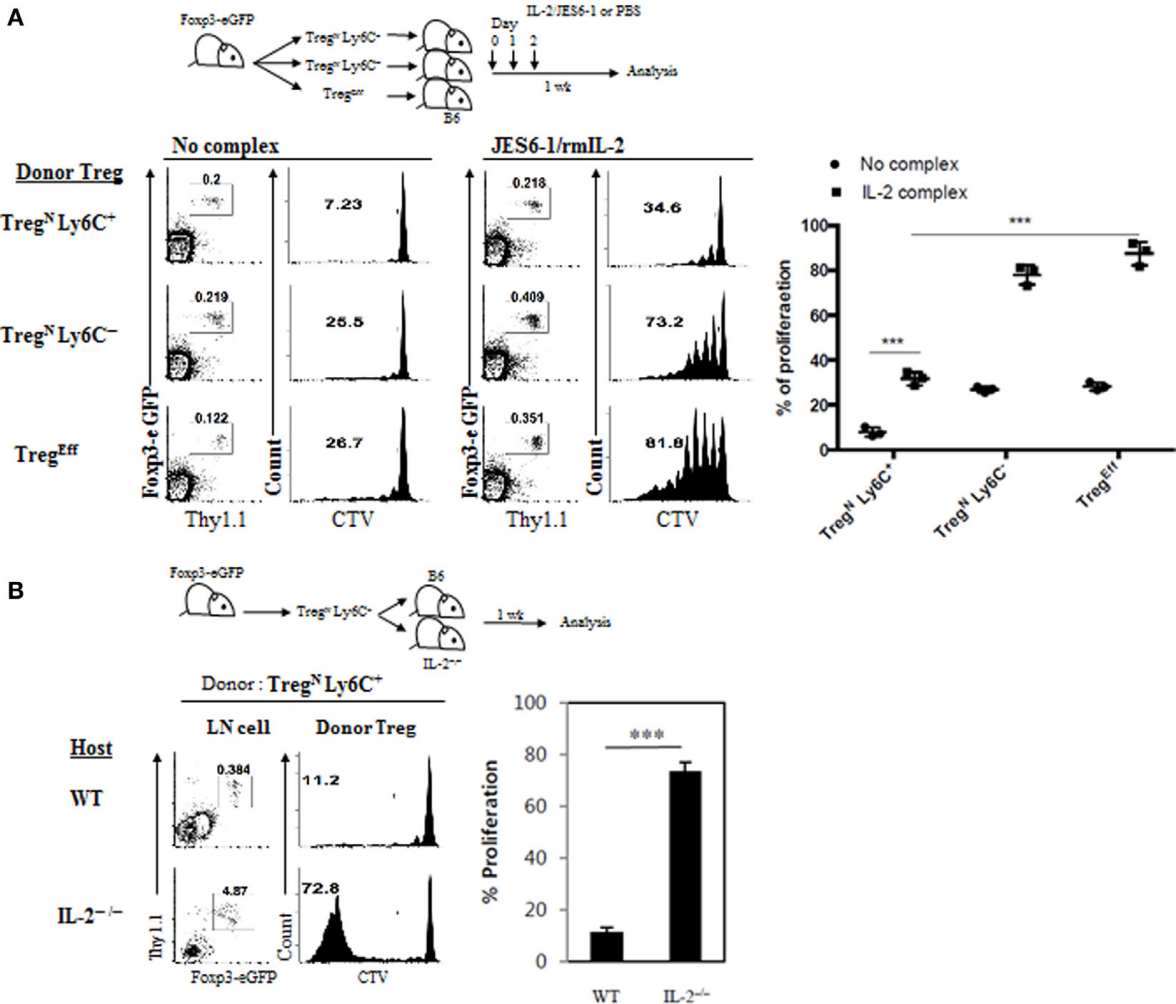
Role of IL-2 in Tconv^Eff^-driven functional recovery of Treg^N^ Ly6C^+^ subset. **(A)** Cell tracker violet (CTV)-labeled T regulatory (Treg) subsets were injected into wild-type (WT) hosts on day 0 and then IL-2/anti-IL-2 complexes were daily injected on days 0–2. Proliferations of donor Treg subsets were analyzed on day 7. **(B)** CTV-labeled Treg subsets were injected into WT or IL-2^−/−^ hosts on day 0 and then donor Treg proliferations were analyzed on day 7. The data are representative of three experiments comprising three mice per group. *p < 0.05, **p < 0.01, and ***p < 0.001.

We next investigated how Tconv^Eff^ populations promote effector conversion and subsequent proliferation of Treg^N^ Ly6C^+^ subset. For this, we sought to test a possibility that the effects by Tconv^Eff^ cells are perhaps mediated indirectly by providing additional homeostatic cues such as IL-2 in particular, as IL-2 is well documented to be essential for many aspects of peripheral Treg homeostasis, including their survival and function (12, 21), and also known to be produced mainly from activated CD4 Tconv^Eff^ populations (21). In fact, all purified Treg subsets (Treg^N^ Ly6C^+^, Treg^N^ Ly6C^−^, and Treg^Eff^) proliferated equally well *in vitro* when responding to exogenous IL-2 and TCR ligation with anti-CD3 (Figure S6A in Supplementary Material), suggesting a potential effect of IL-2 on Treg^N^ Ly6C^+^ subset. To further address the role of IL-2 in converting Treg^N^ subsets *in vivo*, we used a well-established approach of enhancing *in vivo* biological activity of IL-2. For this, a complex form of IL-2 and anti-IL-2 (JES6-1 for Treg-specific activity (22)) was injected into B6 mice that were adoptively transferred with either purified naive (Treg^N^ Ly6C^+^ and Treg^N^ Ly6C^−^) or effector (Treg^Eff^) Treg subsets (Figure 7A, top). In analysis of IL-2-uninjected recipient mice on day 7, both Treg^N^ Ly6C^−^ and Treg^Eff^ donor subsets showed a moderate level of proliferation (~25% by CTV dilution), whereas Treg^N^ Ly6C^+^ subset did not (Figure 7A, bottom left). By contrast, proliferation of all these Treg subsets was significantly increased by administration of IL-2, although the degree of proliferation was far lower for Treg^N^ Ly6C^+^ than for both Treg^N^ Ly6C^−^ and Treg^Eff^ subsets (Figure 7A, bottom right).

Despite moderate enhancement in proliferation by IL-2 injection, the proliferative capacity of Treg^N^ Ly6C^+^ subset was still incomplete with little or no downregulation of Ly6C expression (Figure S6B in Supplementary Material), and the levels were far lower than those of both Treg^N^ Ly6C^−^ and Treg^Eff^ subsets (Figure 7A). This suggest that the effect of injected IL-2 might be insufficient for Treg^N^ Ly6C^+^ subset by a competition with existing host Treg^N^ population whose proliferation was also enhanced by IL-2 (Figure S6C in Supplementary Material). Alternatively, it is also possible that full recovery of proliferation for Treg^N^ Ly6C^+^ subset depends on either additional different cytokines other than IL-2 or mixtures of such various cytokines including IL-2. To directly address a role of endogenous IL-2 *in vivo*, we adoptively transferred purified Treg^N^ Ly6C^+^ subset into either WT or IL-2^−/−^ mice (Figure 7B, top); noted that the latter mice lack Treg development and therefore exhibit uncontrolled activation/expansion of host T cell populations (12). At day 7 after transfer, donor Treg^N^ Ly6C^+^ subset barely proliferated in WT hosts but surprisingly this subset showed a substantial degree of proliferation in IL-2^−/−^ hosts (Figure 7B, bottom). This result therefore suggests that IL-2 alone is perhaps not an essential cytokine cue *per se* but rather other single or combinations of various proinflammatory cytokines, which are likely to be present at relatively high concentrations in hyperlymphoproliferative environment of IL-2^−/−^ hosts, might be involved. In support of this, addition of IL-4, IL-21, and TNF-α to the culture of Treg^N^ Ly6C^+^ subset *in vitro* significantly enhanced their anti-CD3-induced proliferation (Figure S6D in Supplementary Material). Importantly, the enhancing effect of these additional cytokines on the anti-CD3-induced *in vitro* proliferation of Ly6C^+^ Treg cells was largely recapitulated by simple coculture with naive CD4 T cells (Figure S6E in Supplementary Material), further supporting a role of Tconv^Eff^ cells. Collectively, these findings strongly suggest that Tconv^Eff^ cells contribute to enhancing proliferation and conversion of Treg^N^ Ly6C^+^ subset into Treg^Eff^, presumably by providing single or several essential cytokine cue(s).

### Recovery from Poor Immunosuppressive Function of Treg^N^ Ly6C^+^ Subset

**Figure 8:**
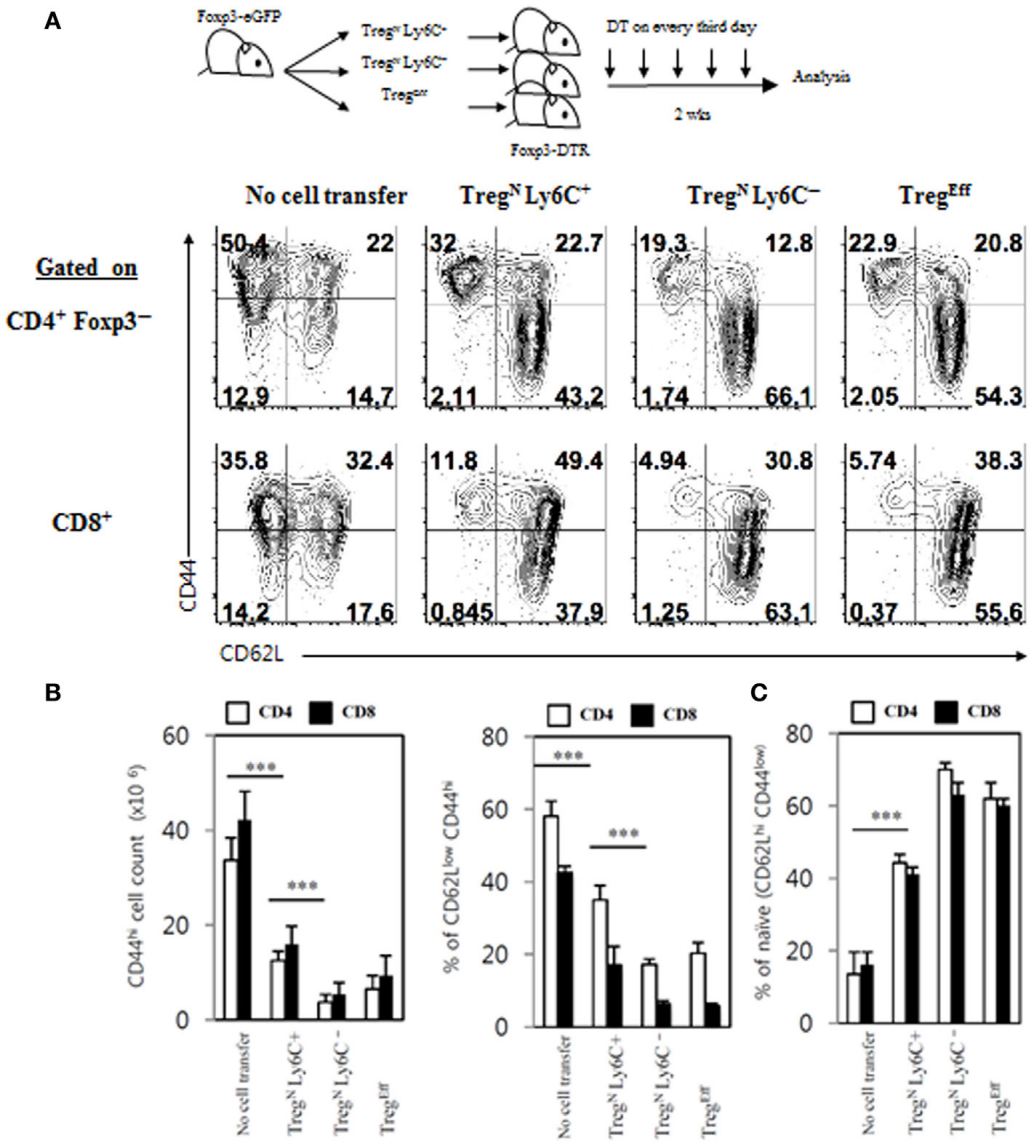
Restoration of immune-suppressive activity of Treg^N^ Ly6C^+^ cells. **(A)** T regulatory (Treg) subsets from Thy1.1^+^ Foxp3-eGFP mice were labeled with cell tracker violet and then injected into wild-type or Foxp3-DTR hosts. The hosts were treated with 1 µg of diphtheria toxin in every third day until day 14. Shown are FACS plots for CD44 and CD62L expressions on host CD4 and CD8 T cells in PLN on day 14 after transfer of Treg subsets. Absolute cell numbers **(B)** and frequencies **(B,C)** for the gated subsets indicated were calculated based on the data shown in **(A)**. The data are representative of three experiments comprising three to five mice per group. *p < 0.05, **p < 0.01, and ***p < 0.001.

Given the previously reported poor immunosuppressive activity of Ly6C^+^ Treg cells *in vitro* and *in vivo* (4), we sought to address a possibility that these cells may regain their suppressive ability with the aid of Tconv^Eff^ cells. To this end, we utilized again the aforementioned Foxp3-DTR mice system to generate an abundant supply of Tconv^Eff^ pools and adoptively transferred purified Treg subsets (Treg^N^ Ly6C^+^, Treg^N^ Ly6C^−^, and Treg^Eff^) into these mice followed by DT treatment (Figure 8, top). Although the expansion of Treg^N^ Ly6C^+^ subset was lower than that of Treg^N^ Ly6C^−^ subset on day 7 (Figure 6B), at day 14 after transfer and DT treatment, we confirmed a similar degree of expansion of all donor Treg subsets in both frequency and numbers (Figure S7A in Supplementary Material). Based on such efficient expansion of donor Treg subsets, we then compared their relative suppressive capacity for preventing host naive T cells from being activated, expanded, and differentiated into conventional effector populations that are CD44^hi^ CD62L^lo^.

As shown in Figure 8A, frequencies of CD44^hi^ CD62L^lo^ CD4 and CD8 effector T cells generated in Foxp3-DTR mice after DT treatment were significantly reduced by adoptively transferred Treg^N^ Ly6C^−^ and Treg^Eff^ subsets, compared to those generated in control DT-treated Foxp3-DTR mice with no Treg cell transfer, which was in agreement with their known suppressive activity reported previously (13). Likewise, Treg^N^ Ly6C^+^ donor subset also showed a significant degree of suppressive effects on the generation of CD44^hi^ CD62L^lo^ effector T cell populations from DT-treated Foxp3-DTR mice, although level of such suppression was slightly less effective than that of Ly6C^−^ Treg subsets (Figures 8A,B). In sharp contrast to the reduced frequency of CD44^hi^ CD62L^lo^ effector T cells, the relative proportion of CD44^lo^ CD62L^hi^ naive CD4 and CD8 T cells in Foxp3-DTR mice after DT treatment was increased by adoptively transferred Treg subsets (Figures 8A,B). In close accord with the strong suppressive activity observed, Treg^N^ Ly6C^+^ donor subset in Foxp3-DTR mice after DT treatment displayed upregulation of various Treg functional markers, such as CTLA-4, LAP, GARP, and GITR, at levels similar to those seen in Treg^N^ Ly6C^−^ and Treg^Eff^ subsets (Figure S7B in Supplementary Material). Besides the enhanced expression of these Treg suppressive markers, Treg^N^ Ly6C^+^ cells in Foxp3-DTR mice after DT treatment also showed the ability to produce immune-suppressive cytokine IL-10, at levels comparable to those seen in Treg^Eff^ cells (Figure S7C in Supplementary Material). Moreover, the Treg^N^ Ly6C^+^ subset in DT-treated Foxp3-DTR mice appeared to gain a full proliferative potential even in a Tconv^Eff^-free environment of irradiated hosts (Figure S8 in Supplementary Material); here, adoptive transfer of Treg^N^ Ly6C^+^ subset purified from day 14 DT-treated Foxp3-DTR mice showed massive proliferation, whereas freshly isolated Treg^N^ Ly6C^+^ counterparts failed to show such proliferation. Collectively, all these findings provide strong evidence that Treg^N^ Ly6C^+^ subset in a “hyperimmune” environment bearing large numbers of Tconv^Eff^ populations can switch their resting naive state into effector state, thereby regaining immune-suppressive activity to maintain self-tolerance.

## Discussion

In this study, we demonstrated that generation and maintenance of Ly6C^+^ Treg cells in the peripheral lymphoid organs are regulated by a unique mechanism distinctly different from those of Ly6C^−^ Treg populations. Consistent with other previous studies, the Ly6C^+^ Treg cells displayed all features characteristics of naive cells in their surface phenotypes, activation status, and functional responses in steady state condition, resulting in a poor degree of proliferative capacity and an impaired immune-suppressive activity. Although Ly6C^+^ Treg subset appeared to be non-functional and gradually lost with augmented apoptotic death with age in the previous studies (4), we demonstrated here that these cells could actively change their phenotype and function from naive to effector cells with a “help” from conventional effector T cell populations. Our findings therefore imply an existence of a homeostatic cross-talk between peripheral Treg pools and conventional effector T cells and highlight a physiological importance of Ly6C^+^ Treg subset for the maintenance of self-tolerance.

The naive phenotypes of Ly6C^+^ Treg subset with a poor functional responsiveness was well defined to be correlated by relatively low degree of TCR self-reactivity (4), which was consistent with the fact that these cells express a slightly reduced level of CD5 and Nur77 compared to those of Ly6C^−^ Treg subsets. It is therefore conceivable that any postthymic Treg precursor cells newly entering into the periphery would have a potential to induce Ly6C and become Ly6C^+^ Treg subset, only if they have a relatively low intrinsic TCR affinity for self-ligands. In fact, a previous study reported, however, that large majority of Ly6C^−^ Treg precursor cells rapidly gained Ly6C expression shortly after adoptive transfer into MHC-II^−/−^ recipient hosts that are lacking self-pMHC ligands (4). This finding suggests that the ability of individual Treg precursor cells to become Ly6C^+^ Treg is perhaps much less dependent on their lower intrinsic TCR affinity for self *per se* but rather largely influenced negatively by a relative abundancy of self-ligands being engaged with any given TCR repertoire. In light of this view, we demonstrated that OT-II CD4 T cells could exhibit a variable capacity to become Ly6C^+^ Treg subset in mixed BM chimera, depending on the relative amounts of Ova presented as a cognate self-antigen. Likewise, we also showed that Ly6C^+^ Treg cell generation from thymic Treg precursor cells transferred into irradiated B6 hosts varied depending on the overall size of the donor cells that are competing for self-ligands. These findings therefore indicate that the potential of inducing Ly6C^+^ Treg is determined in a flexible manner by relative abundancy of self-ligands rather than by intrinsic TCR affinity for them.

If any given TCR can respond to lower amounts of self-ligands and mediate Ly6C^+^ Treg generation from most Treg precursors, one may expect that the impaired proliferative responses of Ly6C^+^ Treg cells is perhaps due to their interactions with too much low levels of cognate self-ligands, resulting from either competition for or scarcity of those self-ligands. This possibility was not the case, however, by the fact that Ly6C^+^ Treg cells from H2M^−/−^ mice, in which all Treg cells are positively selected by only a single self-peptide CLIP from the thymus and maintained by interactions with the same CLIP self-antigen in the periphery, still exhibited a profound defect in their ability to proliferate after adoptive transfer into H2M^−/−^ (on a lymphopenic Rag1^−/−^ background) hosts. These results therefore suggest that once any given Treg precursor cells are induced to differentiate into Ly6C^+^ Treg cells, the resulting population may lose their functional ability to respond to even the same quantity and quality of a cognate self-ligand being engaged in the presence and even in the absence of clonal competition. Although underlying mechanisms remain unclear, this phenomenon observed in Ly6C^+^ Treg cells appeared to resemble activated conventional T cells in an anergic state (23), as was evident for their poor functional responsiveness with impaired *in vivo* proliferation. We demonstrated, however, that unlike those of anergic T cells with a permanent functional failure, the impaired function for Ly6C^+^ Treg cells was not retained in an irreversible manner but rather recovered actively by a certain cue derived from convention effector/memory T cell populations (see below).

Regaining functional responsiveness of Ly6C^+^ Treg cells was apparent in an environment of aged mice in particular. At first glance, the results showing a much lower proportion of Ly6C^+^ Treg cells than Ly6C^−^ CD43^+^ effector Treg cells in the aged mice seemed to reflect a gradual disappearance of the former subset due to their enhanced apoptotic death as reported previously (4). However, we could detect no such obvious enhancement in the apoptosis of Ly6C^+^ Treg cells and instead found much reduced levels of apoptosis (Annexin V^+^ cells) compared to Ly6C^−^ CD43^+^ Treg cells *ex vivo*, perhaps correlating with their increased levels of Bcl-2 expression (18). In an attempt of searching other possible explanation, downregulation of Ly6C expression on Ly6C^+^ Treg cells has been demonstrated in an *in vitro* culture with anti-CD3-induced TCR stimulation although the similar phenomenon has not been addressed *in vivo* (4). Direct evidence as to whether such downregulation indeed occurs *in vivo* and accounts for the substantial reduction of Ly6C^+^ Treg population in aged mice came from the results showing markedly enhanced phenotypic conversion from Ly6C^+^ Treg to Ly6C^−^ Treg after adoptive transfer into an older (14 months old) but not a young (2 months old) recipient host. Moreover, in agreement with the observed alterations in Ly6C expression, the converted Treg cells appeared to be functional with increased proliferative potential and upregulation of various Treg surface markers in these aged hosts. These findings therefore support the above notion that Ly6C^+^ Treg cells are not a permanently “anergic” in their functional status but rather persisted transiently in a “tuned” state perhaps by a unique mechanism that can dynamically modulate their activation threshold for responsiveness to tonic TCR stimuli from self-interactions. An exact mechanism by which the Ly6C^+^ Treg cells are tuned and how they can be relieved from such tuning in a milieu of aged mice will need to be investigated.

With regard to the factors that can promote the age-dependent alterations of Ly6C^+^ Treg cells, we could demonstrate a close relationship of such phenomenon with peripheral abundancy of CD44^hi^ CD62L^lo^ conventional effector T cell populations, a number of which is directly proportional to the age of host mice. In addition to the results from the older mice, this correlation was also evident from two other findings even in a young adult host adoptively transferred with purified Ly6C^+^ Treg: here, enhanced responsiveness of this subset occurred either (1) when cotransferred with conventional CD4 T cells into GF Rag1^−/−^ hosts or (2) when transferred into Foxp3-DTR hosts with DT treatment. Each of these hosts could generate a large number of effector T cell populations and was found to result in substantial increase of proliferative responses of the donor Ly6C^+^ Treg with a concomitant downregulation of Ly6C expression, an upregulation of effector Treg markers, and a functional restoration of immune-suppressive activity. Possible mechanism underlying the impact of such effector populations on Ly6C^+^ Treg is unclear yet nevertheless it seems apparent that this phenomenon could not be explained by IL-2 alone produced by effector T cells, as the phenotypic conversion and increased functional alteration of Ly6C^+^ Treg cells were still observed even in a IL-2-deficient host. Although the data from IL-2^−/−^ mice ruled out the sole effect of IL-2 alone, role of other pro-inflammatory cytokines (either as a single or in combinations) that are abundant in this hyper-immune milieu is still open in question, as we demonstrated the enhancing effects of IL-4, IL-21, and TNF-α on anti-CD3-induced Ly6C^+^ Treg proliferation *in vitro*. These observations with cytokines, together with the similar (and even greater) enhancing effect of coculture of naive CD4 T cells on Treg proliferation *in vitro*, would seem to provide strong support, if not proof, for the possibility that Ly6C^+^ Treg cells are a unique in their property to respond to a certain rise of either single or mixture of cytokines produced from effector T cells. Precisely how the Tcon^Eff^ cells are activated in a manner dependent on specific immune environment of aging or inflammation, and produce sufficient amounts of stimulatory cytokines for naive Treg remains to be addressed. Also, a possibility that the age- or inflammation-dependent effect on naive Treg cells is a simple reflection of their different immune niche is needed to be clarified.

Besides the potential role of cytokine cues in converting functional status of Ly6C^+^ Treg cells, our study does not rule out a role of additional extrinsic factors other than cytokines. For instance, effector T cells might provide a different form of “help” on Ly6C^+^ Treg cells either by a direct T cell–T cell interaction *via* costimulatory molecules, such as CD40L–CD40, or through an indirect modulation of DC activity *via* upregulating ligand molecules for DC–Treg interactions, including self-pMHC as well as CD80, CD86, and ICOSL (24–26). In light of the latter view, it is possible that activation of conventional effector CD4 and/or CD8 T cells may produce various proinflammatory cytokines, which can in turn either activate a steady-state DC or convert a “tolerogenic” DC into stimulatory DC, thereby allowing them to promote functional restoration of Ly6C^+^ Treg cells. Therefore, further investigation as to whether the enhanced DC activation indeed occurs preferentially in the aged mice (and also in IL-2-deficient or DT-treated Foxp3-DTR mice) and whether such effects are independent of commensal microbial and/or dietary food antigens and thus still achievable even in the aged GF or AF mice will be of interest to be performed.

Collectively, all these findings suggest that despite the abovementioned various possible mechanisms, effector T cells play a critical role in rescuing a tuned state of Ly6C^+^ Treg cells. Given that such “detuning” process is accompanied by subsequent alterations in their phenotype and function with regaining immune-suppressive activity, we propose that peripheral Ly6C^+^ Treg cells under steady state may serve as a natural reservoir to supply functional effector Treg cells in a directional (from Ly6C^+^ CD62L^hi^ naive into Ly6C^−^ CD62L^hi^ naive and then into Ly6C^−^ CD62L^lo^ CD43^+^ effector Treg) and also highly regulated manner—which is of particular importance in the aged mice in which thymic Treg generation is severely decreased due to thymic involution. This study therefore provides better understanding for a unique property of Ly6C^+^ Treg population and their role in maintaining self-tolerance.

## Ethics Statement

This research was approved by the Institutional Animal Care and Use Committees (IACUC) of the Pohang University of Science and Technology (2013-01-0012). Mouse care and experimental procedures were performed in accordance with all institutional guidelines for the ethical use of non-human animals in research and protocols from IACUC of the Pohang University of Science and Technology.

## Author Contributions

JL, JK, JY, DK, and HK performed experiments. JL, DH, CS, and JC designed experiments and analyzed and interpreted the data. JS contributed to this study with valuable discussion and critical comments. JL and J-HC wrote the manuscript.

## Conflict of Interest Statement

The authors declare that the research was conducted in the absence of any commercial or financial relationships that could be construed as a potential conflict of interest.
