# Supplementary material for: Phenotypic and Functional Changes of Peripheral Ly6C+ T Regulatory Cells Driven by Conventional Effector T Cells
Source: Front Immunol. 2018 Mar 16;9:437. doi: 10.3389/fimmu.2018.00437 (PMC5864862; doi:10.3389/fimmu.2018.00437)
Supplement: Supplementary file 1 [file data_sheet_1.docx]

Supplementary Material

**Phenotypic and functional conversion of peripheral Ly6C^+^ Treg cells by a cue from conventional effector T cells**

Jun Young Lee^1,2^, JuHee Kim^1,2^, Jaeu Yi^1,2^, Daeun Kim^1,2^, Hee-OK Kim^1,2^, Daehee Han^1,2^, Jonathan Sprent^4-5^, You Jeong Lee^1,2^, Charles D. Surh^1-3^ and Jae-Ho Cho^1,2^.

^1^Academy of Immunology and Microbiology, Institute for Basic Science, Pohang 790-784, Korea.

^2^Department of Integrative Biosciences and Biotechnology, Pohang University of Science and Technology, Pohang 790-784, Korea.

^3^Division of Developmental Immunology, La Jolla Institute for Allergy and Immunology, La Jolla, CA 92037, USA*.*

^4^Immunology Division, Garvan Institute of Medical Research, Darlinghurst, New South Wales 2010, Australia.

^5^St Vincent’s Clinical School, University of New South Wales, Sydney, New South Wales 2010, Australia

**Correspondence:**

Charles D. Surh: csurh@ibs.re.kr

Jae-Ho Cho**:** [jhcho90@ibs.re.kr](mailto:jhcho90@ibs.re.kr)

**Supplementary Figures**

**Figure S1**


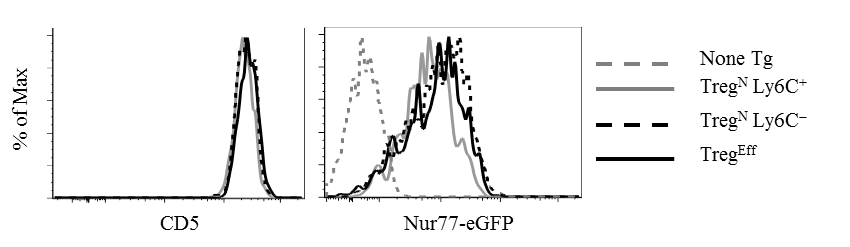


**Figure S1. Ly6C expression on Treg is inversely correlated with TCR strength for self-ligands.** Treg subsets from Nur77-eGFP mice were analyzed for CD5 and Nur77-eGFP expression. Shown are CD5 and Nur77-eGFP expression on PI**^−^** CD4^+^ CD25^+^ Treg subsets.

**Figure S2**


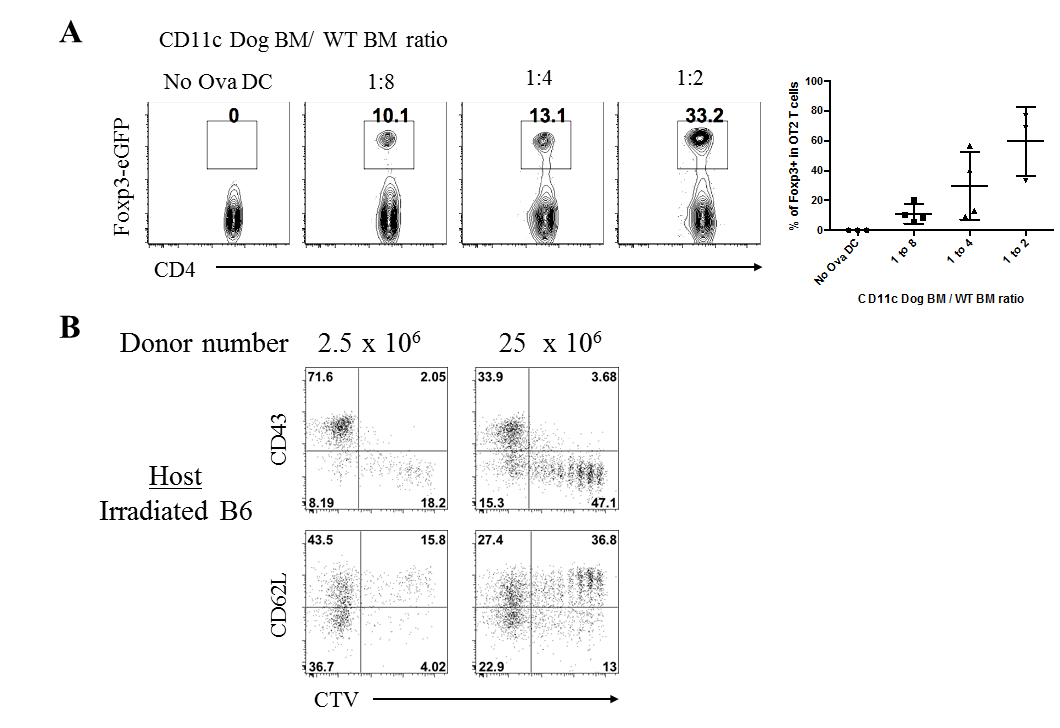


**Figure S2. Effect of self-ag signaling intensity on Treg development and differentiation in the periphery. (**A) Bone marrow chimeras were generated as indicated in Fig 2A. Shown is frequency of Treg in OT-II Thy1.1^+^ CD4^+^ cells in spleen at 8 weeks after BM cell transfer. (B) Cell tracker violet (CTV) labeled, indicated number of CD8-depleted thymocytes from Thy1.1^+^ Foxp3-eGFP mice were injected into irradiated B6 hosts and then proliferation and Ly6C expression were analyzed on day 14 after transfer. Shown are proliferation and the CD43 and CD62L expression on donor Thy1.1^+^ Foxp3^+^ CD4^+^ cells in PLN.

**Figure S3**


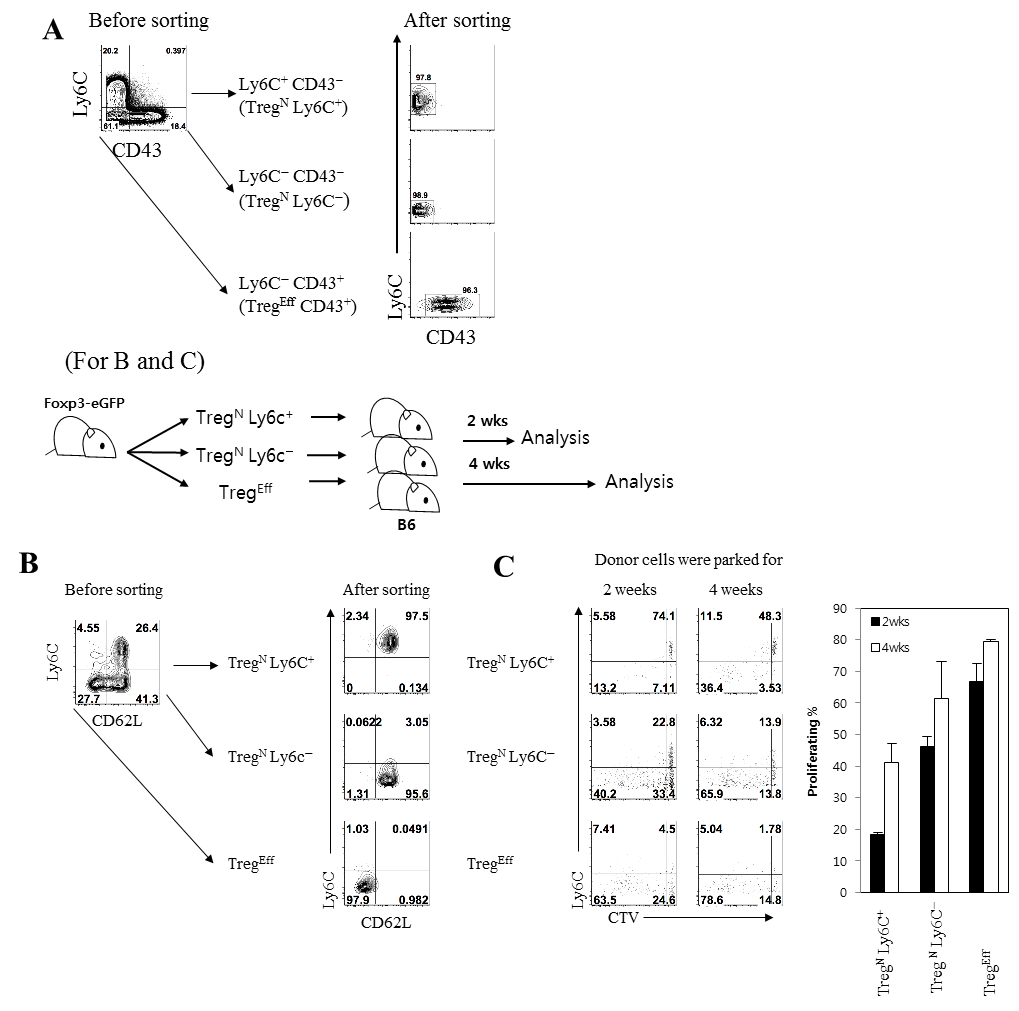


**Figure S3. Sorting strategies and poor responsiveness of Treg^N^ Ly6c^+^ cells in a steady-state normal B6 host.** (A) LN cells from Thy1.1^+^ Foxp3-eGFP mice were depleted for CD4**^−^** cell and then stained with PI, CD4, CD43, and Ly6c. Shown are gated on PI^−^ CD4^+^ eGFP^+^ population. (B) Sorting strategy for Treg^N^ Ly6C^+^, Treg^N^ Ly6C^−^ or Treg^Eff^ subsets. LN cells from Thy1.1^+^ Foxp3-eGFP mice were depleted for CD4^−^ cell and then stained with PI, CD4, CD62L, and Ly6C. Shown are gated on PI^−^ CD4^+^ eGFP^+^ population. (C) CTV labeled Treg subsets were injected into WT hosts and then proliferations of donor Treg subsets were analyzed on day 14 or day 28 after transfer.

**Figure S4**


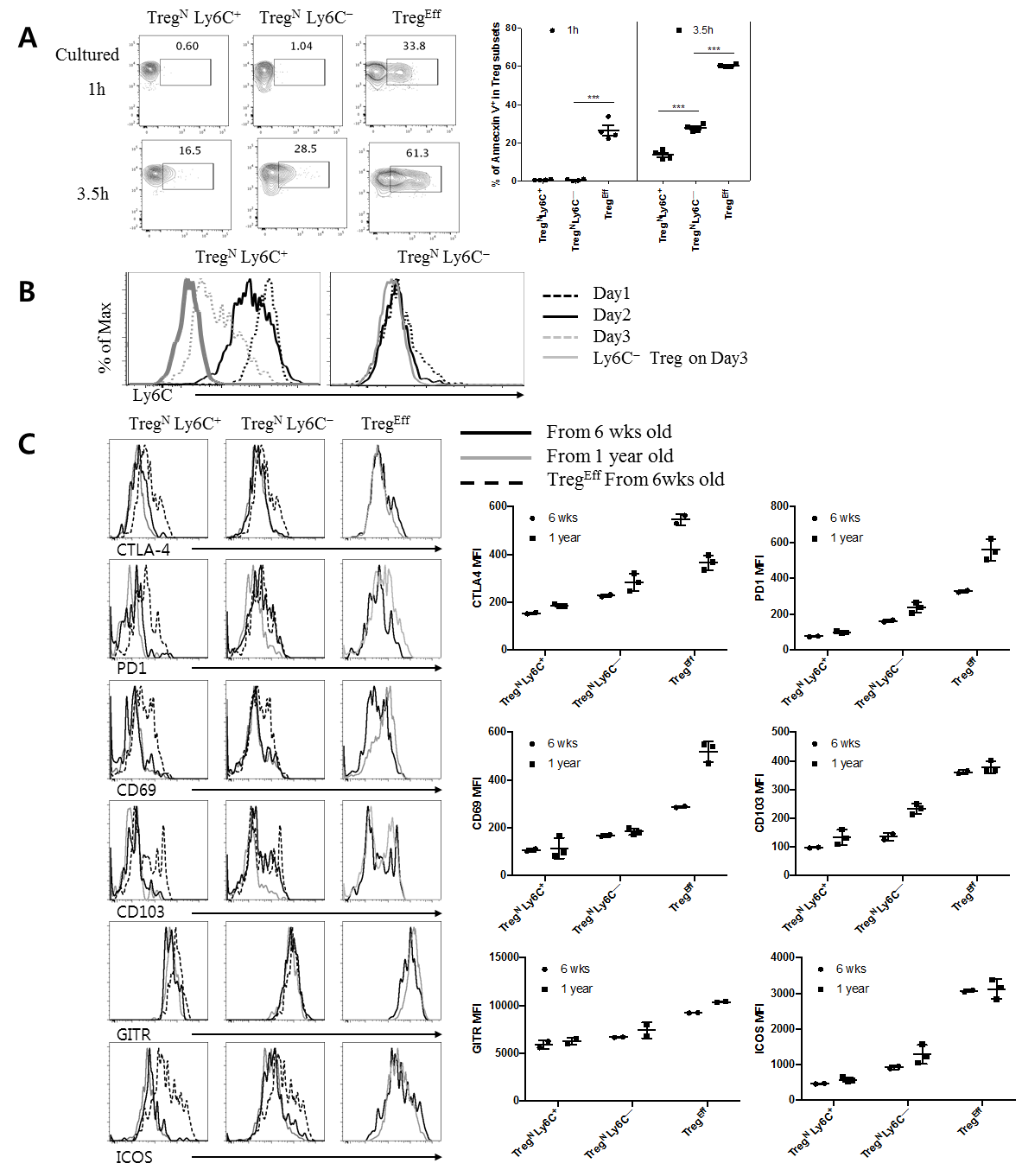


**Figure S4. Decreased Treg^N^ Ly6C^+^ cells proportion in aged host is not associated with preferential apoptosis.** (A) PLN cells from 6-8wks old Foxp3-eGFP mice were cultured for 1 or 3.5hour in the absence of any stimulation. Shown is Annexin V staining on Treg subsets at indicated time points (left) and statistics (right). (B) Treg subsets were stimulated with plate bound anti-CD3 (5 µg / ml) plus anti-CD28 (2.5 µg / ml) for indicated time points. Shown are expressions of Ly6C on cultured Treg subsets at indicated time points. (C) Various marker expressions on Treg subsets from young or aged mice was analyzed.

**Figure S5**

**
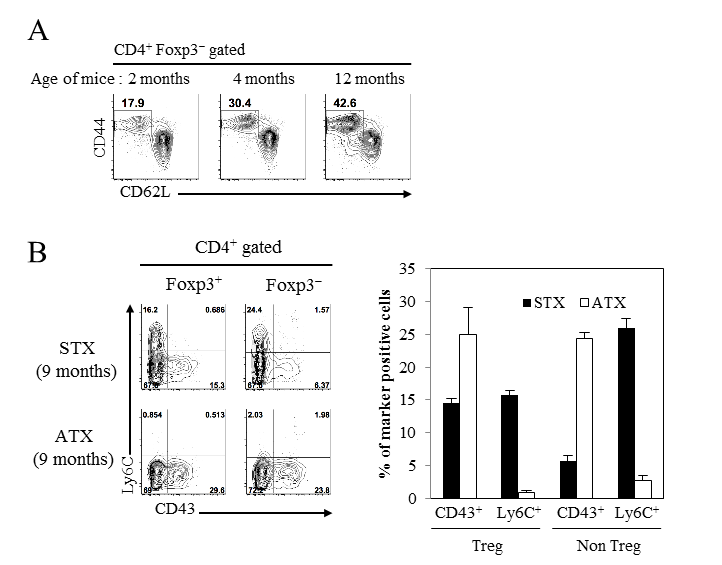
**

**Figure S5. Impact of aging and thymectomy on homeostasis of Tconv^Eff^ and Treg subset.** (A) CD4^+^ Foxp3^−^ cells from differentially aged SPF B6 mice were analyzed for CD62L and CD44 expression. (B) WT mice (6wks old) were thymectomized and then splenic CD4 T cells were analyzed at 9 months later. Shown are FACS plots for Ly6C and CD43 expression on both Foxp3^+^ and Foxp3^−^ CD4^+^ cells (left) and bar graphs for statistics of the analysis (right).

**Figure S6**

**
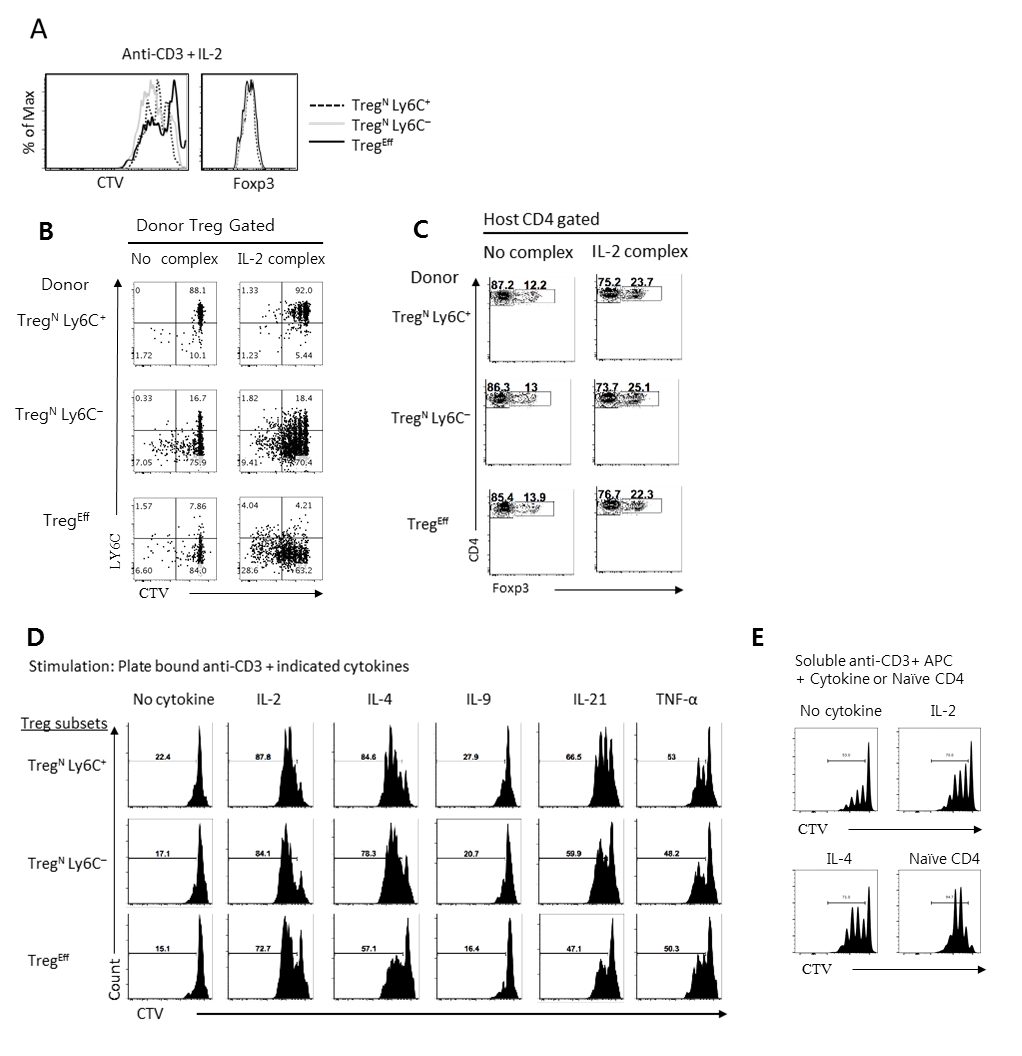
**

**Figure S6. Effect of cytokines on Treg proliferation.** (A) CTV labeled Treg subsets were stimulated with plate bound anti-CD3 (5 µg / ml) plus rmIL-2 (10 ng / ml) for 3 days. Shown are CTV dilution and Foxp3 expression level on cultured cells. (B-C) Mice were treated as described in Fig. 7A. (B) Ly6C expression and proliferation of donor Treg was analyzed. (C) Host Foxp3^+^ Treg expansion was analyzed. (D) CTV labeled Treg subsets were stimulated with plate bound anti-CD3 (5 µg / ml) plus 10 ng / ml of indicated cytokines for 3 days. Shown are proliferations of Treg subsets. (E) Purified Treg^N^ Ly6C+ cells were stimulated with soluble anti CD-3 (2 µg / ml) plus splenocytes from Rag1^−/−^ mice in the presence or absence of IL-2, IL-4 or Naïve CD4 T cells.

**Figure S7**


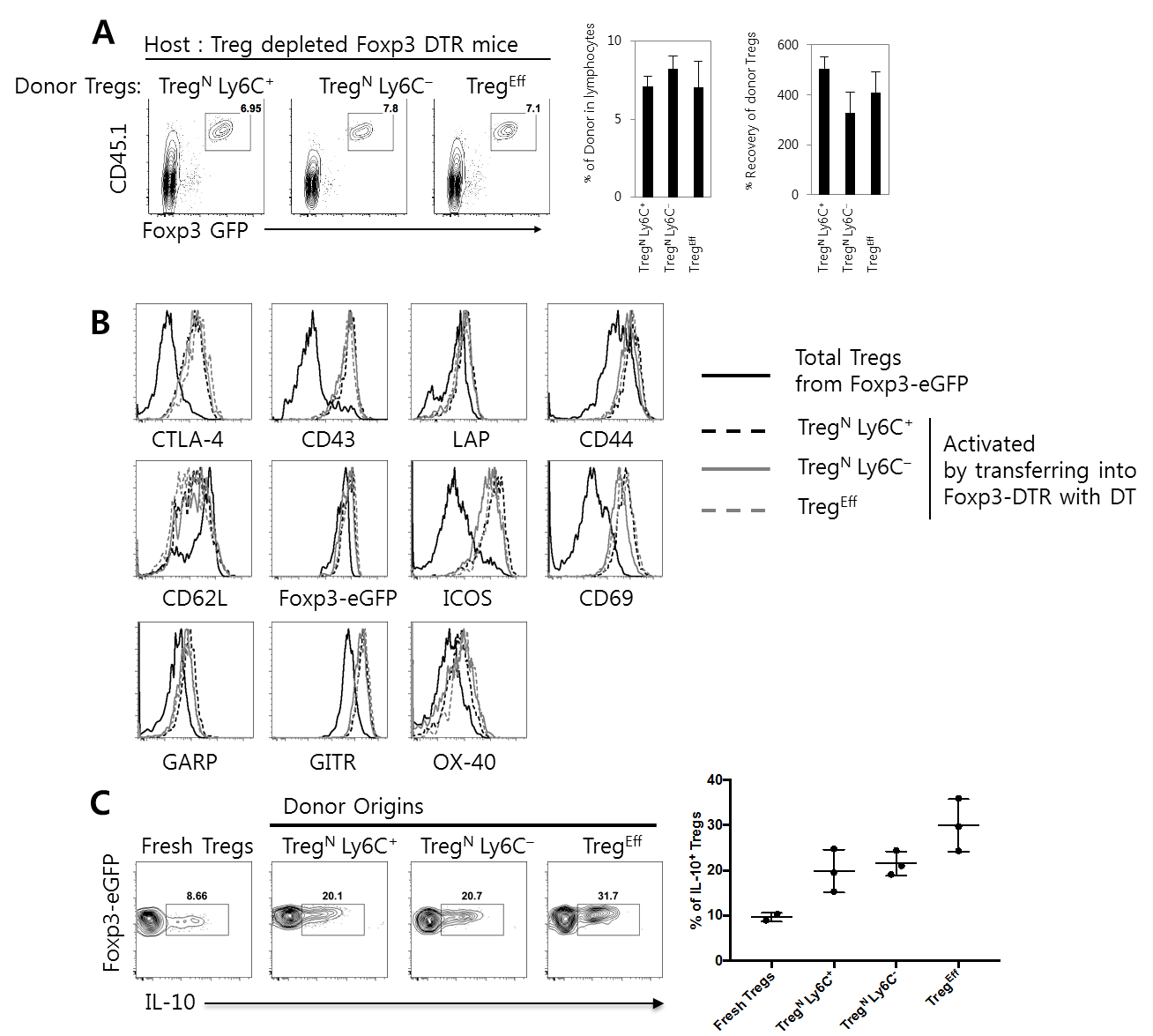


**Figure S7. Each Treg subsets have similar potency to acquire strongly stimulated Treg phenotype under Treg depleted condition.** (A-C) Treg subsets from CD45.1^+^ Foxp3-eGFP or Thy1.1^+^ Foxp3-eGFP mice were transferred into Foxp3-DTR hosts and 1 µg of DT was injected in every third day for 2 weeks. (A) Donor Treg recovery from PLN of Foxp3-DTR mice was shown. (B) Effector marker expressions on *in vivo* stimulated Treg subsets. (C) IL-10 production from strongly activated Treg subsets.

**Figure S8**

**
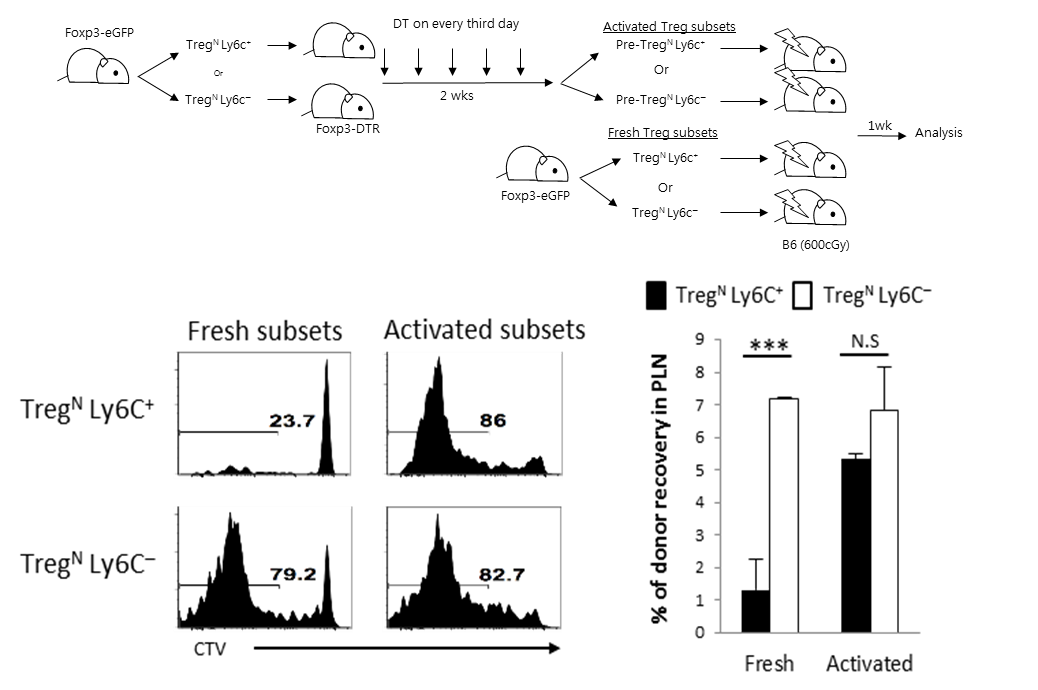
**

**Figure S8. Restoration of proliferative responses of converted Ly6C^+^ Treg subsets**. Treg^N^ Ly6C^+^ or Treg^N^ Ly6C^−^ subsets from Thy1.1^+^ Foxp3-eGFP mice were transferred into Foxp3-DTR hosts followed by injection with 1 µg of DT in every third day for 2 weeks. On day 14, the activated Treg subsets were re-sorted from LN and spleen of Foxp3-DTR hosts and then transferred into irradiated B6 hosts (600 cGy). For control groups, freshly isolated Treg^N^ Ly6C^+^ or Treg^N^ Ly6C^−^ cells were injected into irradiated B6 hosts. Shown are CTV dilution and recovery of Treg subsets in irradiated B6 mice on day 7 after transfer.
